# Unexpected exposure to *Mycobacterium tuberculosis* during bronchoscopy using radial probe endobronchial ultrasound

**DOI:** 10.1371/journal.pone.0246371

**Published:** 2021-01-28

**Authors:** Hyun Sung Chung, Soohyun Bae, Insu Kim, Hyo Yeong Ahn, Jung Seop Eom

**Affiliations:** 1 Department of Internal Medicine, Pusan National University School of Medicine, Busan, Republic of Korea; 2 Department of Internal Medicine, Ulsan University Hospital, University of Ulsan College of Medicine, Ulsan, Republic of Korea; 3 Department of Internal Medicine, Dong-A University Hospital, Busan, Republic of Korea; 4 Department of Thoracic and Cardiovascular Surgery, Pusan National University School of Medicine, Busan, Republic of Korea; 5 Biomedical Research Institute, Pusan National University Hospital, Busan, Republic of Korea; Inha University Hospital, REPUBLIC OF KOREA

## Abstract

**Background:**

Bronchoscopy using radial probe endobronchial ultrasound (EBUS) is performed when a peripheral lung lesion (PLL) is suspected to be malignant. However, pulmonary tuberculosis is diagnosed in some patients, and healthcare workers could therefore be exposed to tuberculosis if sufficient precautions are not taken. In this study, we examined the proportion of and factors associated with unexpected exposure to *Mycobacterium tuberculosis* during bronchoscopy using radial probe EBUS.

**Methods:**

This retrospective study included 970 patients who received bronchoscopy using radial probe EBUS between December 2015 and November 2018. Clinical, histological, radiological, and microbiological data were reviewed.

**Results:**

Pulmonary tuberculosis was diagnosed in 31 patients (3.2%) during bronchoscopy using radial probe EBUS. Patients with a lower age were significantly more likely to be diagnosed with tuberculosis than elderly patients (odds ratio [OR], 0.951; 95% confidence interval [CI], 0.924–0.978; *P* = 0.001). Among the various CT findings, a low HUs difference between pre- and post-enhanced CT (OR, 0.976; 95% CI, 0.955–0.996; *P* = 0.022), the presence of concentric cavitation (OR, 5.211; 95% CI, 1.447–18.759; *P* = 0.012), and the presence of satellite centrilobular nodules (OR, 22.925; 95% CI, 10.556–49.785; *P* < 0.001) were independently associated with diagnosis of tuberculosis.

**Conclusions:**

The proportion of unexpected exposure to *Mycobacterium tuberculosis* during bronchoscopy using radial probe EBUS was 3.2%. A higher risk was independently associated with a younger age and CT findings of a small difference in HUs between pre- and post-enhancement images, concentric cavitation, and the presence of a satellite centrilobular nodule.

## Background

The American College of Chest Physicians and American Association for Bronchology Consensus Statement recommends that healthcare workers should wear an N95 particulate respirator or higher-grade respiratory protection to prevent bronchoscopy-associated *Mycobacterium tuberculosis* infection during bronchoscopy of patients with suspected pulmonary tuberculosis [1]. However, in some patients, pulmonary tuberculosis is diagnosed from bronchoscopy samples when the patient was not initially thought to be suffering from tuberculosis.^2^ Previous studies reported that healthcare workers in the Republic of Korea, which is an intermediate tuberculosis prevalence region (59/100 000 persons per year, 2019), have at least a 4–6% chance of inadvertent exposure to *Mycobacterium tuberculosis* during conventional bronchoscopy [2–4].

Novel bronchoscopy modalities for the diagnosis of peripheral lung lesions (PLLs) have been developed over the past 20 years, and include electromagnetic navigational bronchoscopy and virtual bronchoscopy navigation [5]. In particular, bronchoscopy using radial probe endobronchial ultrasound (EBUS) is considered to be a reasonable diagnostic approach with an acceptable diagnostic yield and low complication rate [6–8]. Although bronchoscopy using radial probe EBUS is generally performed when lung cancer is suspected, previous studies reported that bronchoscopy samples obtained using radial probe EBUS resulted in a final diagnosis of pulmonary tuberculosis in 3.9–11.0% of patients [9–15].

Because of the widespread use of low-dose computed tomography (CT) for lung cancer screening in high risk patients, the use of radial probe EBUS bronchoscopy for the diagnosis of PLLs has also increased [16,17]. However, little is known about the unexpected exposure to *Mycobacterium tuberculosis* during radial probe EBUS bronchoscopy for PLLs when lung malignancy is initially suspected. Therefore, we performed a multicenter cross-sectional study to identify the proportion of unexpected exposure to *Mycobacterium tuberculosis* during bronchoscopy using radial probe EBUS, and to identify factors associated with an increased probability of it.

## Methods

### Study population

This retrospective study was conducted using the bronchoscopy databases of Pusan National University Hospital (a university-affiliated tertiary referral hospital in Busan, Republic of Korea) and Ulsan University Hospital (a university-affiliated secondary referral hospital in Ulsan, Republic of Korea) for the period December 2015 to November 2018. During the study period, 993 patients with PLL (797 and 196 patients at Pusan National University and Ulsan University Hospitals, respectively) received bronchoscopy using radial probe EBUS. Among them, 23 patients were excluded because lung cancer was previously confirmed and the radial probe EBUS bronchoscopy was performed as a re-biopsy for the identification of T790M mutation of epidermal growth factor receptor [18]. This left 970 patients for selection for the study. Some of the clinical data on the patients enrolled between 2015 and 2018 was included in two articles published in 2018 and 2019 [8,19]. This study was approved by the institutional review boards of Pusan National University Hospital (IRB no. 1906-033-080) and Ulsan University Hospital (IRB no. 2020-07-011). All data were fully anonymized and the ethics committee waived the requirement for informed consent. All patients’ medical records were followed-up from the time they received radial probe EBUS to December 2019.

### Analysis of the CT scans

PLL was defined as an intrapulmonary lesion existing beyond the segmental bronchus that is generally invisible on conventional bronchoscopy [20]. The mean diameter of a PLL was defined as the average of its maximum and vertical diameter. A positive bronchus sign was defined as the presence of a bronchus leading directly to the PLL [21]. The margin of the PLL was classified as follows [22,23]: 1) smooth when the margin was well-demarcated with round or oval-shape curves; 2) lobulated when the margin was distinguished by some smooth and relatively large convexities; 3) spiculated when the margin was irregular and had multiple radiating strands; and 4) pneumonic consolidation when the margin could not be distinguished because of surrounding consolidation. PLLs were classified as solid, part-solid, or ground-glass opacity according to a visual assessment method based on CT attenuation [24]. The distance from the pleura was measured as the closest distance between the PLL and the visceral pleura. The Hounsfield units (HU) of the PPL on pre- and post-enhancement CT phases were measured using mediastinal window images to minimize volume averaging [25].

If the lesion showed cavitation, the maximal thickness of the cavity wall was measured, and it was classified as concentric cavitation if the wall thickness was uniform, or eccentric cavitation otherwise [26]. The following accompanying CT findings were also analyzed: 1) presence of a satellite centrilobular nodule; 2) bronchiectasis; 3) anthracofibrosis of the airway; 4) pulmonary emphysema; 5) fibrocalcific tuberculosis; 6) interstitial lung diseases; 7) atelectasis; and 8) pleural effusion (S1 Appendix).

### Bronchoscopic procedure and specimens

All patients underwent bronchoscopy with a 4.0-mm-sized flexible bronchoscope (BF-P260F; Olympus, Tokyo, Japan) after conscious sedation with midazolam and fentanyl. For local anesthesia, 2% lidocaine was injected into the tracheobronchial tree via the working channel of the bronchoscope. After airway inspection, the bronchoscope was advanced as far as possible into the bronchus of the target lesion under CT image guidance. Thereafter, a 20-MHz radial probe EBUS (UM-S20-17S; Olympus, Tokyo, Japan) covered by a guide sheath (K-201; Olympus, Tokyo, Japan) was advanced through the working channel until resistance was met. Then, under X-ray fluoroscopic guidance, the radial probe EBUS was pulled back slightly to acquire the ultrasound images [10]. Radial probe EBUS images were classified as: 1) within; 2) adjacent; or 3) invisible, as described previously [14]. Ultrasound image analysis was performed according to the classifications of Kurimoto *et al*. (S1 Table). When the location of the target lesion was identified, the radial probe was retrieved with the guide sheath being kept in place. Brushing cytology and a forceps biopsy were then performed through the guide sheath under fluoroscopic guidance. After obtaining the cytology and biopsy samples, the guide sheath was removed and bronchial washing of the target lesion was performed through the working channel with 5 ml of sterile saline.

The bronchial washing fluids were used to perform an acid-fast bacillus smear with culture and a real-time polymerase chain reaction (PCR) for mycobacterium. Fluorescence microscopy with auramine-rhodamine staining was used for the acid-fast bacillus smear. Both solid (3% Ogawa medium) and liquid medium (BACTEC MGIT 960 system; Becton Dickinson Microbiology Systems, Sparks, MD) were used for the mycobacterium culture. A real-time PCR for mycobacterium (AdvanSure TB/NTM real-time PCR kit; LG Life Science, Seoul, Republic of Korea) was also performed on the bronchial washing fluid.

If a final diagnosis could not be determined from the bronchoscopic samples, an additional percutaneous needle biopsy or surgical biopsy was performed. When the pathological findings of the percutaneous needle biopsy or surgical biopsy specimens were suspicious for tuberculosis, such as the presence of chronic granulomatous inflammation, PCR (MTB-PCR kit; Biosewoom, Seoul, Republic of Korea) was additionally performed on the tissue specimen, at the discretion of the pathologist.

### Diagnosis of pulmonary tuberculosis

Tuberculosis was diagnosed as follows: 1) *Mycobacterium tuberculosis* was cultured from the bronchial washing fluid; and 2) PCRs for *Mycobacterium tuberculosis* using either the bronchial washing fluid or tissue specimen were positive and clinicoradiological improvements were achieved after standard anti-tuberculosis treatment [27] and 3) compatible histological findings, such as chronic granulomatous inflammation with clinicoradiological correlations.

### Statistical analysis

Statistical analyses were performed using R version 3.6.6 (R-Project, GNU GPL). All results are presented as number and percentage for categorical variables and median with interquartile range [IQR] for continuous variables. Data comparisons were made using χ2 or Fisher’s exact tests for categorical variables, and independent *t*-tests or Wilcoxon rank-sum tests for continuous variables. Multivariate logistic regression analysis was performed using factors with a *P*-value < 0.1 in the univariate analysis, to identify independent factors related to unexpected exposure to *Mycobacterium tuberculosis*. *P*-values < 0.05 were considered statistically significant. Receiver operating characteristics curves were plotted to calculate the area under the curve, Youden index, sensitivity, and specificity.

## Results

### Baseline characteristics

The baseline characteristics of the 970 patients are shown in Table 1. The median age was 69 years (IQR, 61–76) and 62.4% of patients were of male gender. The most frequent PLL margin type on CT was a lobulated type, which was shown in 450 patients (46.4%). Solid lesions were the most common opacity, being found in 812 patients (83.7%). The median mean diameter of the PLLs was 27.1 mm (IQR, 19.4–37.8 mm), and the median distance from the pleura was 8.0 mm (IQR, 0.0–20.1 mm). One hundred and twenty patients (12.4%) had cavity formation, and 22 (18.3%) of these had concentric cavitation. A positive bronchus sign on CT was found in 898 patients (92.5%). The most frequent accompanying CT finding was pulmonary emphysema, which occurred in 266 patients (27.4%), followed by anthracofibrosis in 154 patients (15.8%), atelectasis in 103 patients (10.6%), and fibrocalcific tuberculosis in 99 patients (10.2%; S2 Table).

**Table 1:** Baseline characteristic of the 970 study patients.

Variables	No. (%), or median (interquartile range)
Age, years	69 (61–76)
Male gender	605 (62.4)
Location	
Right upper lobe	271 (27.9)
Right middle lobe	73 (7.5)
Right lower lobe	231 (23.8)
Left upper lobe	248 (25.2)
Left lower lobe	147 (15.2)
Margin of peripheral lung lesion	
Smooth	161 (16.6)
Lobulated	450 (46.4)
Spiculated	266 (27.4)
Pneumonic consolidation	93 (9.6)
CT Opacity	
Solid	812 (83.7)
Mixed	140 (14.4)
Ground-glass opacity	18 (1.9)
Mean diameter, mm	27.1 (19.4–37.8)
Distance from pleura, mm	8.0 (0.0–20.1)
Difference in Hounsfield unit*	28.7 (12.7–46.3)
Cavity formation	120 (12.4)
Cavity wall thickness, mm	14.8 (8.0–19.1)
Concentric cavitation^†^	22/120 (18.3)
Positive bronchus sign on CT	898 (92.5)
Endobronchial ultrasound images	
Within lesion	807 (83.0)
Adjacent to lesion	137 (14.1)
Invisible lesion	26 (2.7)

*Differences in Hounsfield units between pre- and post-enhancement images were measured in 817 patients (84.2%).

^†^Data are presented as No./total (%).

### Overall diagnosis

Pulmonary tuberculosis was diagnosed in 31 patients (3.2%) who received bronchoscopy using radial probe EBUS (Table 2). Ultrasound image analyses are summarized in S1 Table. *Mycobacterium tuberculosis* was cultured from the bronchial washing specimens of 28 patients (90.3%), and the PCR for mycobacterium was positive in 25 patients (80.6%). Culture and PCR for mycobacterium using the bronchial washing fluid were both positive in 19 patients (61.3%). Using the surgical specimen, PCR for *Mycobacterium tuberculosis* was positive in four patients (12.9%), and all were diagnosed with pulmonary tuberculosis with compatible histological findings. In four patients with negative results for both culture and PCR, the histologic samples showed chronic granulomatous inflammation without necrosis. Because there was no clinical or radiological evidence of tuberculosis, all these patients were followed-up without any treatment. There were no changes in the size and characteristics of the lung lesions after more than 6 months of follow-up, and consequently, all the lesions were regarded as benign lesions other than tuberculosis. Two patients had both lung cancer and pulmonary tuberculosis. Otherwise, lung cancer was diagnosed in 691 patients (71.2%), followed by benign disease such as organizing pneumonia and nontuberculous mycobacterium lung disease in 35 patients (3.6%), and metastatic cancer from an extrathoracic malignancy such as colon cancer, breast cancer, or lymphoma in 24 patients (2.5%; Table 3).

**Table 2:** Diagnostic methods in the 31 patients with pulmonary tuberculosis.

Diagnostic method	No. (%)
Bronchial washing specimen	
*Mycobacterium tuberculosis*-positive culture	28 (90.3)
PCR positive for mycobacterium	25 (80.6)
Tissue specimen	
PCR positive for mycobacterium	4 (12.9)

PCR = polymerase chain reaction.

**Table 3:** Overall diagnoses of the 970 study patients.

Diagnosis	No. (%)
Malignant disease	
Lung cancer*	693 (71.4)
Colon cancer	5 (0.5)
Breast cancer	4 (0.4)
Sarcoma	3 (0.3)
Lymphoma	3 (0.3)
Thyroid cancer	2 (0.2)
Malignant melanoma	1 (0.1)
Renal cell carcinoma	1 (0.1)
Mesothelioma	1 (0.1)
PEComatous tumor	1 (0.1)
Cervical cancer	1 (0.1)
Bladder cancer	1 (0.1)
Endometrial cancer	1 (0.1)
Benign disease	
Pulmonary tuberculosis*	31 (3.2)
Nontuberculous mycobacteria	10 (1.0)
Organizing pneumonia	10 (1.0)
Necrotizing pneumonia	4 (0.4)
Aspergilloma	4 (0.4)
Bronchiolitis obliterans organizing pneumonia	2 (0.2)
Sarcoidosis	1 (0.1)
Cryptococcus	1 (0.1)
Fungal infection	1 (0.1)
Parasite infection	1 (0.1)
Eosinophilic granulomatosis with polyangiitis	1 (0.4)
Unknown	189 (19.5)

*Two patients had both lung cancer and pulmonary tuberculosis simultaneously.

### Factors associated with pulmonary tuberculosis

Univariate analysis showed that the patients with pulmonary tuberculosis were significantly younger than those without pulmonary tuberculosis (61 yrs *vs*. 68 yrs, *P* = 0.004; Table 4). There was a significant difference in the PPL margin between patients with and without tuberculosis (*P* = 0.007). The difference in HUs between pre- and post-enhancement images was significantly lower in patients with pulmonary tuberculosis than in those without tuberculosis (15.0 *vs*. 29.0, *P* = 0.026). In patients with a cavitary lesion, the proportion of lesions showing concentricity was significantly higher in those patients with tuberculosis than in those without tuberculosis (50.0% *vs*. 16.7%, *P* = 0.040). Patients with pulmonary tuberculosis had a higher probability of having a satellite centrilobular nodule on CT than those without pulmonary tuberculosis (67.7% *vs*. 7.2%, *P* < 0.001).

**Table 4:** Data of the patients with and without pulmonary tuberculosis.

Variables	With pulmonary tuberculosis (n = 31)	Without pulmonary tuberculosis (n = 939)	*P*-value
Age, years	61 (49–72)	68 (61–76)	0.004
Male gender	22 (70.9)	583 (62.1)	0.737
Location			0.635
Right upper lobe	8 (25.8)	263 (28.0)	
Right middle lobe	2 (6.5)	71 (7.6)	
Right lower lobe	5 (16.1)	226 (24.1)	
Left upper lobe	9 (29.0)	239 (25.5)	
Left lower lobe	7 (22.5)	140 (14.9)	
Margin			0.007
Smooth	6 (19.3)	155 (16.5)	
Lobulated	7 (22.5)	443 (47.2)	
Spiculated	11 (35.4)	255 (27.2)	
Pneumonic consolidation	7 (22.6)	86 (9.2)	
CT opacities			0.307
Solid	29 (93.5)	783 (83.3)	
Mixed	1 (3.2)	139 (14.8)	
Ground-glass opacity	1 (3.2)	17 (1.8)	
Mean diameter, mm	26.5 (17.3–45.9)	27.2 (19.7–37.7)	0.799
Distance from pleura, mm	5.4 (0.0–18.3)	8.0 (0.0–20.1)	0.409
Difference in Hounsfield units*	15.0 (3.3–44.4)	29.0 (13.0–46.4)	0.026
Cavity formation	6 (19.4)	114 (12.1)	0.446
Concentric cavitation^†^	3/6 (50.0)	19/114 (16.7)	0.040
Positive bronchus sign on CT	31 (100.0)	867 (92.2)	0.521
Endobronchial ultrasound images			0.488
Within lesion	25 (80.6)	782 (83.2)	
Adjacent to lesion	4 (12.9)	133 (14.2)	
Invisible lesion	2 (6.5)	24 (2.6)	
Companion CT findings			
Satellite centrilobular nodule	21 (67.7)	68 (7.2)	< 0.001
Bronchiectasis	2 (6.5)	35 (3.7)	0.823
Anthracofibrosis	1 (3.2)	153 (16.3)	0.070
Pulmonary emphysema	7 (22.5)	259 (27.5)	0.538
Fibrocalcific tuberculosis	6 (19.3)	93 (9.9)	0.212
Interstitial lung disease	0 (0.0)	35 (3.7)	0.512
Atelectasis	2 (6.5)	101 (10.7)	0.564
Pleural effusion	4 (12.9)	79 (8.4)	0.668

*Differences in Hounsfield units between pre- and post-enhancement images were measured in 817 patients (84.2%).

^†^Data are presented as No./total (%).

Multivariate logistic regression analysis was conducted to identify independent factors associated with unexpected exposure to *Mycobacterium tuberculosis* (Table 5). The results revealed that a younger age was significantly associated with a higher chance of tuberculosis diagnosis (odds ratio [OR], 0.951; 95% confidence interval [CI], 0.924–0.978; *P* = 0.001). Among the various CT findings, a lower difference in HUs between pre- and post- enhancement images (OR, 0.976; 95% CI, 0.955–0.996; *P* = 0.022), the presence of concentric cavitation (OR, 5.211; 95% CI, 1.447–18.759; *P* = 0.012), and the presence of satellite centrilobular nodules (OR, 22.925; 95% CI, 10.556–49.785; *P* < 0.001) were independently associated with an unexpected exposure to *Mycobacterium tuberculosis* during bronchoscopy using radial probe EBUS.

**Table 5:** Logistic regression analysis to identify factors associated with pulmonary tuberculosis.

Variables	Odds ratio (95% confidence interval)	*P*-value
Age (per year)	0.951 (0.924–0.978)	0.001
Margin		
Smooth *vs*. Lobulated	0.412 (0.136–1.244)	0.116
Smooth *vs*. Spiculated	1.122 (0.407–3.093)	0.825
Smooth *vs*. Pneumonic consolidation	2.116 (0.689–6.479)	0.190
Difference in Hounsfield units* (per digit)	0.976 (0.955–0.996)	0.022
Concentric cavitation^†^	5.211 (1.447–18.759)	0.012
Satellite centrilobular nodule	22.925 (10.556–49.785)	< 0.001
Bronchiectasis	0.836 (0.111–6.303)	0.862
Anthracofibrosis	0.171 (0.023–1.265)	0.084
Fibrocalcific tuberculosis	2.183 (0.873–5.459)	0.095

*Differences in Hounsfield units between pre- and post-enhancement images were measured in 817 patients (84.2%).

^†^In patients with and without tuberculosis, cavitary lesions were found in 114 and 31 patients, respectively.

**Fig 1:**
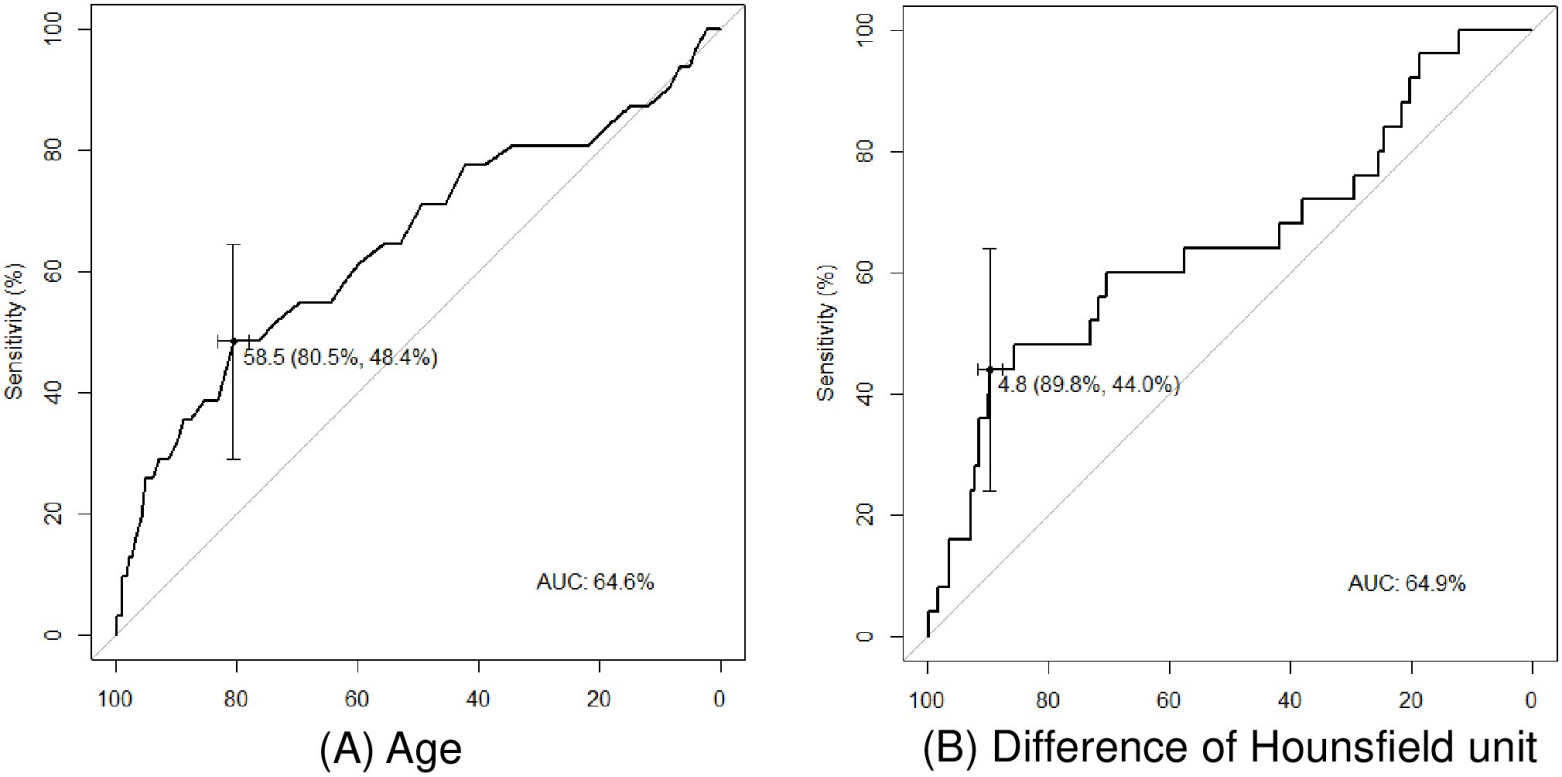
Receiver operating characteristics (ROC) curves for predicting unexpected exposure to *Mycobacterium tuberculosis*. (A) ROC curve of age. (B) ROC curve of the difference in Hounsfield units between pre- and post-enhancement images.

Receiver operating characteristics curves based on age and the difference in HUs between pre- and post-enhancement images are shown in Fig 1. For age and the difference in HUs, the areas under the curve were 64.6 (sensitivity 48.4%; specificity 80.5%) and 64.9 (sensitivity 44.0%; specificity 89.8%), respectively. Analysis of the Youden’s index indicated that the optimal cut-off values for age and the difference in HUs for predicting unexpected exposure to *Mycobacterium tuberculosis* were 58.5 and 4.8, respectively.

## Discussion

Previously, we found that pulmonary tuberculosis was unexpectedly diagnosed in 4.6% of patients when conventional bronchoscopy was performed, and that the risk factors associated with an unexpected diagnosis of tuberculosis on conventional bronchoscopy were anthracofibrosis, bronchiectasis, or atelectasis on chest CT [2]. In this study, the proportion of unexpected exposure to *Mycobacterium tuberculosis* during radial probe EBUS bronchoscopy for PLL was 3.2%. Our results indicate that healthcare workers in the bronchoscopy suite could be accidentally exposed to *Mycobacterium tuberculosis* during bronchoscopy using radial probe EBUS if high-grade respiratory precautions, such as the use of an N95 particulate respirator, are not undertaken. In addition, we identified several risk factors associated with an unexpected exposure to *Mycobacterium tuberculosis*, such as age and distinct CT findings (low difference in HUs, concentric cavitation, and the presence of a satellite centrilobular nodule).

During conventional bronchoscopy, the probability of unexpected diagnosis of pulmonary tuberculosis is known to range from 0.3% to 1.3% in low pulmonary tuberculosis prevalence regions, and 3.7% to 9.1% in intermediate or high prevalence regions [2]. Previous studies on bronchoscopy using radial probe EBUS showed various rates of tuberculosis diagnosis ranging from 3.9–11.0%, irrespective of the prevalence of tuberculosis (Table 6). In their multicenter prospective study conducted in Japan, Oki *et al*. reported a 3.9% rate of tuberculosis on bronchoscopy for small lung nodules less than 3 cm [9], while Herth *et al*. reported results for bronchoscopy using radial probe EBUS performed in 2002 and 2006 in Germany [12,14], and found that the proportions of tuberculosis were quite different between the two years (4% *vs*. 11%, respectively). Our results suggest that the proportion of tuberculosis diagnosed on radial probe EBUS varies depending on the physician’s case selection, that it is not directly related to the regional prevalence of tuberculosis, and that the risk of healthcare personnel being exposed to *Mycobacterium tuberculosis* during bronchoscopy using radial probe EBUS is underestimated, with it being as high as that on conventional bronchoscopy.

**Table 6:** Diagnosis of pulmonary tuberculosis in previous studies on radial probe endobronchial ultrasound bronchoscopy.

Authors	Year	Region	Design	No. lesions	No. tuberculosis
Herth et al. [12]	2002	Germany	Prospective	50	2 (4.0%)
Shirakawa et al. [11]	2004	Japan	Prospective	50	2 (4.0%)
Kurimoto et al. [10]	2004	Japan	Prospective	150	12 (8.0%)
Herth et al. [14]	2006	Germany	Prospective	54	6 (11.0%)
Huang et al. [13]	2009	Taiwan	Retrospective	83	5 (6.0%)
Tamiya et al. [15]	2013	Japan	Prospective	68	3 (4.4%)
Oki et al. [9]	2015	Japan	Prospective	305	12 (3.9%)

**Fig 2:**
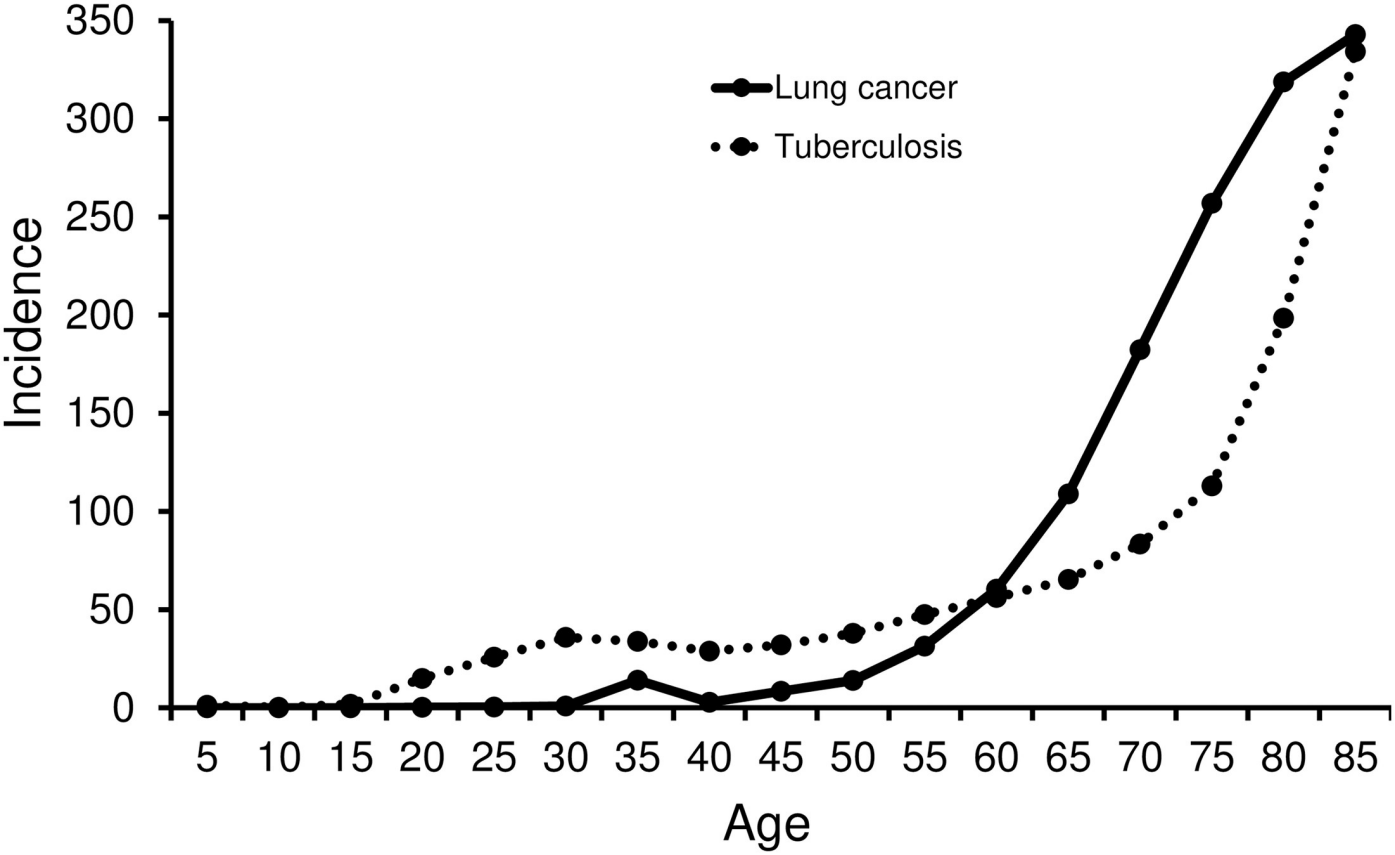
Comparison of the incidence of tuberculosis and lung cancer according to age in the Republic of Korea (100 000 persons per year).

Our results indicate that patient age is one of the predicting factors for an unexpected exposure to *Mycobacterium tuberculosis* during bronchoscopy using radial probe EBUS, with patients younger than 58.5 years being more likely to have pulmonary tuberculosis. Previous epidemiological data show differences in age distribution between tuberculosis and lung cancer. Fig 2 shows a graph comparing the incidence of tuberculosis with that of lung cancer using data from the Korea Centers for Disease Control and Prevention [4,28]. The incidence of tuberculosis was similar from 30s to 50s, and then, it rapidly increases after 60s. The incidence of lung cancer is relatively low before 50s, whereas, it increases explosively after 60s. Taken together, before 60s, the incidence of tuberculosis is approximately twice higher than those of lung cancer. In addition, pulmonary tuberculosis is known to be one of the important differential diagnoses of PLLs, comprising more than 3% of all patients. Accordingly, our results suggest that the risk of a patient with a peripheral lung nodule being diagnosed with tuberculosis is significantly higher in younger patients than in elderly patients.

Kim et al [29]. reported that the presence of a satellite centrilobular nodule and a difference in HUs of less than 20 between pre- and post-enhancement images were useful predictors for benign nodules rather than malignant lesions, whereas Lee et al [30]. identified that the presence of concentric cavitation could suggest the possibility of tuberculosis. In a similar context, we verified that a small difference in HUs (less than 4.8), concentric cavitation, and the presence of a satellite centrilobular nodule on CT were significantly associated with unexpected exposure to *Mycobacterium tuberculosis* in certain patient populations who received radial probe EBUS. Moreover, two patients were finally diagnosed with both lung cancer and tuberculosis. Our results suggest that even if lung cancer is strongly suspected on CT, if these risk factors associated with tuberculosis are present on CT scan, healthcare workers should be prepared for possible exposure to *Mycobacterium tuberculosis*.

This study has several limitations. First, the retrospective design of the study may have resulted in selection bias. Second, contrast-enhanced CT was performed on 817 patients (84.2%); therefore, the difference in HU between pre- and post-enhancement images was not evaluated for all patients. Third, all the data were obtained from the Republic of Korea, which is an intermediate tuberculosis prevalence region, and it may therefore be difficult to generalize the study results to other regions. Finally, the degree of infectivity associated with small peripheral tuberculous lesions is unclear. Generally, patients with a small tuberculosis lesion have low infectivity; however, the bronchoscopy procedure produces lots of respiratory droplets. We believe that healthcare workers should be aware of the risk and protect themselves from any possibility of tuberculosis infection during bronchoscopy with radial probe EBUS. To verify our results, a prospective study with a large study population recruited from multiple regions is needed.

## Conclusions

We found that the proportion of unexpected exposure to *Mycobacterium tuberculosis* during radial probe EBUS bronchoscopy was 3.2%. A higher risk was independently associated with a younger patient age (less than 58.5 years) and distinct CT findings of a small difference in HUs between pre- and post-enhancement images (less than 4.8), concentric cavitation, and the presence of a satellite centrilobular nodule.

## Supporting information

S1 Table Ultrasound image analyses of patients with pulmonary tuberculosis. (DOCX)

S2 Table Accompanying CT findings of the 970 patients who received bronchoscopy using radial probe endobronchial ultrasound. (DOCX)

S1 Appendix Accompanying CT findings. (DOCX)

## Abbreviations

CI: confidence interval
CT: computed tomography
EBUS: endobronchial ultrasound
HU: Hounsfield unit
IQR: interquartile range
OR: odds ratio
PCR: polymerase chain reaction
PLL: peripheral lung lesion
