# Supplementary material for: Unexpected exposure to Mycobacterium tuberculosis during bronchoscopy using radial probe endobronchial ultrasound
Source: PLoS One. 2021 Jan 28;16(1):e0246371. doi: 10.1371/journal.pone.0246371 (PMC7843011; doi:10.1371/journal.pone.0246371)
Supplement: S1 Table — (DOCX) [file pone.0246371.s001.docx]

S1 Table. Ultrasound image analyses of patients with pulmonary tuberculosis.

| Type of ultrasound image analyses | | No. (%) |
| --- | --- | --- |
| I, homogeneous pattern | Ia, with patent vessels and patent bronchioles | 2 (6.5) |
|  | Ib, without vessels and bronchioles | 15 (48.4) |
| II, hyperechoic dots and linear arcs pattern | IIa, without vessels | 7 (22.6) |
|  | IIb, with patent vessels | 1 (3.2) |
| III, heterogeneous pattern | IIIa, with hyperechoic dots and short lines | 2 (6.5) |
|  | IIIb, without hyperechoic dots and short lines | 4 (12.9) |
