# Supplementary material for: Unexpected exposure to Mycobacterium tuberculosis during bronchoscopy using radial probe endobronchial ultrasound
Source: PLoS One. 2021 Jan 28;16(1):e0246371. doi: 10.1371/journal.pone.0246371 (PMC7843011; doi:10.1371/journal.pone.0246371)
Supplement: S2 Table — (DOCX) [file pone.0246371.s002.docx]

S2 Table. Accompanying CT findings of the 970 patients who received bronchoscopy using radial probe endobronchial ultrasound.

| Accompanying CT findings | No. (%) |
| --- | --- |
| Satellite centrilobular nodule | 89 (9.2) |
| Bronchiectasis | 37 (3.8) |
| Anthracofibrosis | 154 (15.8) |
| Pulmonary emphysema | 266 (27.4) |
| Fibrocalcific tuberculosis | 99 (10.2) |
| Interstitial lung disease | 35 (3.6) |
| Atelectasis | 103 (10.6) |
| Pleural effusion | 83 (8.6) |
