# Supplementary material for: Unexpected exposure to Mycobacterium tuberculosis during bronchoscopy using radial probe endobronchial ultrasound
Source: PLoS One. 2021 Jan 28;16(1):e0246371. doi: 10.1371/journal.pone.0246371 (PMC7843011; doi:10.1371/journal.pone.0246371)
Supplement: S1 Appendix — (DOCX) [file pone.0246371.s003.docx]

S1 Appendix. Accompanying CT findings.

A satellite centrilobular nodule was defined as a well-circumscribed accessory focus clearly separated from the main lesion [1]. Bronchiectasis was defined as a visible bronchus in the outer one-third of the lung or a diameter of the inner lumen greater than that of the accompanying pulmonary artery [2]. Anthracofibrosis of the airway was defined as smooth luminal narrowing of a bronchus, surrounded by calcified or noncalcified lymph nodes in elderly patients without a relevant history of pneumoconiosis or smoking [3]. Pulmonary emphysema was defined as a low-attenuation lung area lacking a distinct wall because of structural destruction [4]. Fibrocalcific parenchymal pulmonary tuberculosis was defined as old fibrocalcific changes of healed tuberculosis [5]. Interstitial lung disease was defined as typical imaging findings of reticular opacities, honeycombing, or ground-glass opacity, predominantly in the peripheral and basal lung areas [6]. Atelectasis was defined as increased attenuation of the affected lung associated with reduced lung volume [4]. Pleural effusion was defined as a concave or convex-shaped fluid density within the visceral and parietal pleural layers [7].

**References**

1. Deslauriers J, Brisson J, Cartier R, Fournier M, Gagnon D, Piraux M, Beaulieu M. Carcinoma of the lung. Evaluation of satellite nodules as a factor influencing prognosis after resection. J Thorac Cardiovasc Surg. 1989;97(4):504-12.

2. Koh W-J, Lee KS, Kwon OJ, Jeong YJ, Kwak S-H, Kim TS. Bilateral bronchiectasis and bronchiolitis at thin-section CT: diagnostic implications in nontuberculous mycobacterial pulmonary infection. Radiology. 2005; 235(1): 282-8.

3. Kim HY, Im JG, Goo JM, Kim JY, Han SK, Lee JK, Song JW. Bronchial anthracofibrosis (inflammatory bronchial stenosis with anthracotic pigmentation): CT findings. AJR Am J Roentgenol. 2000; 174(2): 523-7.

4. Hansell DM, Bankier AA, MacMahon H, McLoud TC, Muller NL, Remy J. Fleischner Society: glossary of terms for thoracic imaging. Radiology. 2008;246(3):697-722.

5. Medlar EM. The pathogenesis of minimal pulmonary tuberculosis; a study of 1,225 necropsies in cases of sudden and unexpected death. Am Rev Tuberc. 1948;58(6):583-611.

6. Misumi S, Lynch DA. Idiopathic pulmonary fibrosis/usual interstitial pneumonia: imaging diagnosis, spectrum of abnormalities, and temporal progression. Proc Am Thorac Soc. 2006;3(4):307-14.

7. Abramowitz Y, Simanovsky N, Goldstein MS, Hiller N. Pleural effusion: characterization with CT attenuation values and CT appearance. AJR Am J Roentgenol. 2009;192(3):618-23.
