# Supplementary material for: Unexpected exposure to Mycobacterium tuberculosis during bronchoscopy using radial probe endobronchial ultrasound
Source: PLoS One. 2021 Jan 28;16(1):e0246371. doi: 10.1371/journal.pone.0246371 (PMC7843011; doi:10.1371/journal.pone.0246371)
Supplement: Attachment — Submitted filename: Response to reviewer comment.docx [file pone.0246371.s004.docx]

Thank you for inviting us to revise and re-submit our manuscript for publication in *PLOS ONE*. We have addressed the concerns raised by the reviewers in a point-by-point format. The English language was also professionally checked before this resubmission of the article. All changes have been marked in red font in the revised manuscript. We hope that the changes meet with your approval.

**# Response to the comments of Editor**

**▪ Comment 1**

Current clinical guidelines recommend clinicians to do protection against M tuberculosis during bronchoscopy for patients suspected of having M tuberculosis or not. Therefore, it would be very helpful if they describe what the difference in risk for M tuberculosis is between routine bronchoscopy and radial probe EBUS.

**▪ Response**

We agree that a description of the difference in risk factors between conventional bronchoscopy and radial probe endobronchial ultrasound (EBUS) is important for preventing healthcare workers from acquiring pulmonary tuberculosis in the bronchoscopy suite. In general, radial probe EBUS is performed for histological confirmation when lung cancer is clinically suspected through a chest CT scan. Accordingly, most radial probe EBUS results are confirmed as lung malignancy, but a few cases are unexpectedly diagnosed as pulmonary tuberculosis instead of lung cancer. Previous studies demonstrated rates of 3.9–11% for unexpected pulmonary tuberculosis diagnosis on radial probe EBUS [1,2]. However, the current guidelines recommend that bronchoscopists use an N95 mask or higher-grade respiratory precaution when mycobacterial infection is suspected [3].

We found that unexpected diagnosis of tuberculosis on radial probe EBUS is associated with a young patient age, the presence of satellite nodules, the presence of concentric cavitation, and a low Hounsfield unit difference between the pre- and post-enhanced CT. Most patients who received radial probe EBUS had a small lung nodule less than 3 cm, and accordingly, the unexpected diagnosis of pulmonary tuberculosis showed certain differences in risk factors between conventional bronchoscopy and radial probe EBUS. We previously published an article on “Exposure to *Mycobacterium tuberculosis* during conventional bronchoscopy in patients with unexpected tuberculosis”, and found that the presence of anthracofibrosis, bronchiectasis, or atelectasis on chest CT were independently associated with unexpected pulmonary tuberculosis [4]. To clarify the differences in risk factors associated with the unexpected diagnosis of pulmonary tuberculosis, we have modified the Discussion section as follows:

1. See page 11, lines 8 to 11

“Previously, we found that pulmonary tuberculosis was unexpectedly diagnosed in 4.6% of patients when conventional bronchoscopy was performed, and that the risk factors associated with an unexpected diagnosis of tuberculosis on conventional bronchoscopy were anthracofibrosis, bronchiectasis, or atelectasis on chest CT [2].”

2. See page 11, lines 13 to 16

“Our results indicate that healthcare workers in the bronchoscopy suite could be accidentally exposed to *Mycobacterium tuberculosis* during radial probe EBUS if they do not take high-grade respiratory precautions, such as using an N95 particulate respirator.”

**References**

1. Am J Rrespir Crit Care Med. 2015; 192(4): 468-476.

2. Chest. 2006; 129(1): 147-150.

3. Chest. 2005; 128(3): 1742-1755.

4. PloS one. 2016; 11(5): e0156385.

**▪ Comment 2**

In addition, they need to explain the differences between the radiological finding that they suggested as a suspected M tuberculous infection and the current knowledge or the novelity of the radiological finding that they suggested.

**▪ Response**

Previous radiological studies found that the incidental discovery of pulmonary tuberculosis is associated with the presence of centrilobular nodules, cavitation, and low HU differences between pre- and post-enhanced CT. In a similar context, we identified and confirmed the risk factors for unexpected diagnosis of pulmonary tuberculosis in a patient population who received radial probe EBUS. We believe that our results will remind bronchoscopists about the radiological risk factors for an unexpected diagnosis of pulmonary tuberculosis when they perform radial probe EBUS.

In addition, we found that a younger age was highly related to an unexpected diagnosis of pulmonary tuberculosis. Our results indicate that patients younger than 58.5 years are more likely to have pulmonary tuberculosis than lung malignancy. We believe that international readers will consider ‘age’ as the simplest and easiest patient characteristic for predicting the possibility of incidental exposure to *Mycobacterium tuberculosis* in the bronchoscopy suite. To improve the clarity of our findings, we have modified the Discussion section as follows (see page 12, lines 25 to page 13, lines 4): “In a similar context, we verified that a small difference in HUs (less than 4.8), concentric cavitation, and the presence of a satellite centrilobular nodule on CT were significantly associated with unexpected exposure to *Mycobacterium tuberculosis* in certain patient populations who received radial probe EBUS.”

**▪ Comment 3**

In the ethics statement in the manuscript and in the online submission form, please provide additional information about the patient records used in your retrospective study, including: a) whether all data were fully anonymized before you accessed them and/or whether the IRB or ethics committee waived the requirement for informed consent; b) the date range (month and year) during which patients' medical records were accessed.

**▪ Response**

To make ethics statement clear, we have modified the Methods as follows (see page 5, lines 19 to 21): “All data were fully anonymized and the ethics committee waived the requirement for informed consent. All patients’ medical records were followed-up from the time they received radial probe EBUS to December 2019.”

**▪ Comment 4**

Thank you for stating the following in the Acknowledgments Section of your manuscript:

'This work was supported by Department of Biostatistics, Biomedical Research Institute,

Pusan National University Hospital.'

We note that you have provided funding information that is not currently declared in your Funding Statement. However, funding information should not appear in the Acknowledgments section or other areas of your manuscript. We will only publish funding information present in the Funding Statement section of the online submission form.

Please remove any funding-related text from the manuscript and let us know how you would like to update your Funding Statement. Currently, your Funding Statement reads as follows:

'The authors received no specific funding for this work.'

**▪ Response**

We agree that the acknowledgements were a little ambiguous. The ‘Department of Biostatistics, Biomedical Research Institute’ did not provide funds, but did help with statistical analysis, especially calculation of the Youden index and receiver operating characteristics curves. We have changed the Acknowledgements section as follows (see page 17, lines 3 to 4): “The Department of Biostatistics, Biomedical Research Institute, Pusan National University Hospital provided help with the statistical analysis performed in the present study”

**# Response to the comments of Reviewer 1**

**▪ Comment 1**

The manuscript has not clearly stated objectives to guide the focus of the study.

**▪ Response**

We agree that the description of the objectives of the present study was somewhat unclear. In general, radial probe endobronchial ultrasound (EBUS) is performed for histological diagnosis when lung cancer is clinically suspected on CT scan. Accordingly, most radial probe EBUS results are confirmed as lung malignancy, although a few cases are unexpectedly diagnosed as pulmonary tuberculosis instead of lung cancer. Previous studies demonstrated rates of 4–11% for unexpected pulmonary tuberculosis diagnosis on radial probe EBUS, irrespective of the prevalence of tuberculosis [1-4]. However, the current guideline recommends that bronchoscopists use an N95 mask or higher-grade respiratory precaution only when mycobacterial infection is suspected clinically or radiologically [5].

We previously published an article on “Exposure to *Mycobacterium tuberculosis* during conventional bronchoscopy in patients with unexpected tuberculosis”, which revealed that the presence of anthracofibrosis, bronchiectasis, or atelectasis on CT scan were independently associated with unexpected diagnosis of pulmonary tuberculosis [6]. However, there is little known about the unexpected diagnosis of pulmonary tuberculosis during peripheral bronchoscopy involving techniques such as radial probe EBUS, virtual bronchoscopy navigation, or electromagnetic navigation bronchoscopy in patients with a small lung nodule. After the introduction of the National Lung Screening Trial (NLST) [7], the number of patients with small nodules on low-dose screening CT abruptly increased. Consequently, the use of peripheral bronchoscopy for the pathologic diagnosis of small nodules also increased. We therefore believe that it is important for pulmonary physicians to know how many patients are unexpectedly diagnosed with pulmonary tuberculosis on radial probe EBUS, and to know the relevant factors associated with its occurrence and how to prevent infection. Our results should inform and remind healthcare workers of the possibility of unexpected exposure to *Mycobacterium tuberculosis* when performing bronchoscopy using radial probe EBUS. To further clarify our objectives, we have modified the Background section as follows (see page 4, lines 22 to see page 5, lines 3): “However, little is known about the unexpected diagnosis of pulmonary tuberculosis during radial probe EBUS bronchoscopy for PLLs when lung malignancy is initially suspected. Therefore, we performed a multicenter cross-sectional study to identify the incidence of unexpected diagnoses of pulmonary tuberculosis and the factors associated with an increased probability of it during bronchoscopy using radial probe EBUS.”

**References**

1. Am J Rrespir Crit Care Med. 2015; 192(4): 468-476.

2. Chest. 2006; 129(1): 147-150.

3. Respirology. 2009; 14(6): 859-864.

4. Eur Respir J. 2002; 20(4): 972-974.

5. Chest. 2005; 128(3): 1742-1755.

6. PloS one. 2016; 11(5): e0156385.

7. N Engl J Med. 2011;365:395-409.

**# Response to the comments of Reviewer 2**

**▪ Comment 1**

Since the country of the study setting is moderately endemic with Tuberculosis, then pulmonary tuberculosis is a major differential diagnosis in such study candidates, and unexpected diagnosis of TB is a statement that cannot be accepted.

**▪ Response**

We thank you for pointing out a very important issue in the present study. This study was conducted in the Republic of Korea, which is an intermediate tuberculosis prevalence region (59/100 000 persons per year, 2019) [1], and we found that the incidence of unexpected diagnosis of pulmonary tuberculosis during bronchoscopy using endobronchial ultrasound (EBUS) was 3.2%. Previous studies on bronchoscopy using radial probe EBUS showed various rates of tuberculosis diagnosis, irrespective of the prevalence of tuberculosis. In their multicenter prospective study conducted in Japan, Oki *et al.* reported a 3.9% rate of tuberculosis on bronchoscopy for small lung nodules less than 3 cm [2]. Herth *et al*. reported results for bronchoscopy using radial probe EBUS performed in 2002 and 2006 in Germany [3,4]. Interestingly, the proportions of tuberculosis found differed considerably between 2002 and 2006 (4% *vs.* 11%, respectively). From previous studies, it can be deduced that the proportion of tuberculosis diagnosis in radial probe EBUS procedures varies depending of the physician’s case selection, regardless of where and when radial probe EBUS is performed. To clarify the description of the regional prevalence of tuberculosis and the unexpected diagnosis rate during radial probe EBUS, we have modified the Discussion section as follows (see page 11, lines 21 to see page 12, lines 8): “Previous studies on bronchoscopy using radial probe EBUS showed various rates of tuberculosis diagnosis ranging from 3.9–11.0%, irrespective of the prevalence of tuberculosis (Table 6). In their multicenter prospective study conducted in Japan, Oki *et al.* reported a 3.9% rate of tuberculosis on bronchoscopy for small lung nodules less than 3 cm [9], while Herth *et al*. reported results for bronchoscopy using radial probe EBUS performed in 2002 and 2006 in Germany [12,14], and found that the proportions of tuberculosis were quite different between the two years (4% *vs.* 11%, respectively). Our results suggest that the proportion of tuberculosis diagnosed on radial probe EBUS varies depending on the physician’s case selection, that it is not directly related to the regional prevalence of tuberculosis, and that the risk of healthcare personnel being exposed to *Mycobacterium tuberculosis* during bronchoscopy using radial probe EBUS is underestimated, with it being as high as that on conventional bronchoscopy.”

**References**

1. Korea Centers for Disease Control and Prevention; 2020:218-219.

2. Am J Rrespir Crit Care Med. 2015; 192(4): 468-476.

3. Eur Respir J. 2002; 20(4): 972-974.

4. Chest. 2006; 129(1): 147-150.

**▪ Comment 2**

Retrospective study is a study design applied to cohort studies while the used design is record based cross-sectional study.

**▪ Response**

We agree that this study is of a cross-sectional design. Electronic medical records of all study subjects were reviewed from the time they received radial probe EBUS to December 2019. We have changed the description of the study design as follows (see page 4, lines 22 to page 5, lines 3): “Therefore, we performed a multicenter cross-sectional study to identify the incidence of unexpected diagnoses of pulmonary tuberculosis during bronchoscopy using radial probe EBUS, and to identify factors associated with an increased probability of it.”

**▪ Comment 3**

The conclusion is too long and has a divergent conclusion which is the recommendation to use better protection inside bronchoscopy room which is not accepted for two reasons:

1- This conclusion is not related to the aim of the study
2- Infection control measure in bronchoscopy room ideally should protect from respiratory infections including tuberculosis, in addition, the context of the study did not assess how much personnel adhered to these measure.

**▪ Response**

3-1. We acknowledge that there was too much information in the conclusion, which could confuse the reader. We have revised the Conclusion according to the objectives of this study as follows:

Abstract (see page 2, lines 19 to 23): “The incidence of unexpected diagnosis of tuberculosis during bronchoscopy using radial probe EBUS was 3.2%. A higher risk was independently associated with a younger age and CT findings of a small difference in HUs between pre- and post-enhancement images, concentric cavitation, and the presence of a satellite centrilobular nodule.”

Conclusions part (see page 11, lines 22 to page 14, lines 1): “We found that the incidence of unexpected diagnosis of pulmonary tuberculosis during radial probe EBUS bronchoscopy was 3.2%. A higher risk was independently associated with a younger patient age (less than 58.5 years) and distinct CT findings of a small difference in HUs between pre- and post-enhancement images (less than 4.8), concentric cavitation, and the presence of a satellite centrilobular nodule.”

3-2. . Generally, radial probe EBUS is performed when early-stage lung cancer is suspected on CT scan. The current guideline recommends that bronchoscopists use an N95 mask or higher-grade respiratory precaution only when mycobacterial infection is suspected according to clinical or radiological indications [1]. Therefore, radial probe EBUS is generally performed following standard precautions using an anti-droplet mask, gloves, and gown, without higher-grade respiratory precautions such as an N95 particulate respirator. Likewise, all the radial probe EBUS procedures investigated in our study were performed following standard respiratory precautions. Consequently, our results suggest that even if lung cancer is strongly suspected on CT scan, healthcare workers should be prepared for possible exposure to *Mycobacterium tuberculosis* if risk factors associated with an unexpected diagnosis of tuberculosis are present. To clarify the description of respiratory precautions, we have made changes to the Discussion as follows (see page 11, lines 13 to 16): “Our results indicate that healthcare workers in the bronchoscopy suite could be accidentally exposed to *Mycobacterium tuberculosis* during bronchoscopy using radial probe EBUS if high-grade respiratory precautions, such as the use of an N95 particulate respirator, are not undertaken.”

**References**

1. Chest. 2005; 128(3): 1742-1755.

**# Response to the comments of Reviewer 3**

**▪ Comment 1**

The diagnostic yield (predictive values) of EBUS depends on the prevalence of tuberculosis in a community. The culture and PCR are not absolutely excluding the diagnosis of TB and sometimes the clinician must rely on histopathologic description of tissue.

**▪ Response**

We agree that tuberculosis can also be diagnosed with compatible pathologic findings. In our study, four histologic samples showed chronic granulomatous inflammation, although there was no necrosis in the granuloma. None of these patients were considered to be suspicious for tuberculosis on initial CT scan, and all were followed up without any treatment. There were no changes in the size and characteristics of the lung lesions after more than 6 months of follow-up, and consequently all lesions were regarded as benign lesions other than tuberculosis.

Generally, a thin bronchoscope (usually less than 4 mm in size) can be introduced near to peripheral lung lesions, and then a 1.4-mm radial EBUS probe is advanced through the working channel of the bronchoscope to precisely identify the peripheral lung lesion. Therefore, specimens for culture and PCR are directly collected from the lung lesion, which differs from the generally performed procedure of bronchial washing. We believe that the reliability of specimens collected for culture and PCR by radial probe EBUS was high, and that accordingly, no patient with tuberculosis was diagnosed by pathologic findings alone. To clarify the diagnostic procedure for tuberculosis, we have revised the Methods and Results as follows:

Method part (see page 8, lines 5 to 7)

“and 3) compatible histological findings, such as chronic granulomatous inflammation with clinicoradiological correlations.”

Results part (see page 9, lines 17 to 23)

“In four patients with negative results for both culture and PCR, the histologic samples showed chronic granulomatous inflammation without necrosis. Because there was no clinical or radiological evidence of tuberculosis, all these patients were followed-up without any treatment. There were no changes in the size and characteristics of the lung lesions after more than 6 months of follow-up, and consequently, all the lesions were regarded as benign lesions other than tuberculosis.”

**▪ Comment 2**

Regarding the risk of transmission, when a patient is sputum-negative case for AFB, the risk of transmission during the procedure is not really very high, although the bronchoscopist should be aware of transmission-based precautions.

**▪ Response**

We agree that the risk of tuberculosis transmission is low if a sputum acid-fast bacilli smear is negative. Unfortunately, a sputum acid-fast bacilli smear was not routinely obtained from the study patients as they were initially suspected to have a lung malignancy. Generally, patients with a small tuberculosis lesion, such as all the subjects in the present study, have low infectivity; however, the bronchoscopy procedure produces lots of respiratory droplets. We believe that healthcare workers should be aware of the risk and protect themselves from any possibility of tuberculosis infection. The relationship between the degree of infectivity and peripheral lung lesions should be verified in a future prospective study, and we have added a discussion on infectivity to the Discussion section as follows (see page 13, lines 13 to 18): “Finally, the degree of infectivity associated with small peripheral tuberculous lesions is unclear. Generally, patients with a small tuberculosis lesion have low infectivity; however, the bronchoscopy procedure produces lots of respiratory droplets. We believe that healthcare workers should be aware of the risk and protect themselves from any possibility of tuberculosis infection during bronchoscopy with radial probe EBUS.”

**▪ Comment 3**

As a clinician I am eager to hear about the sonographic features of tuberculosis and mimicking process by EBUS including coagulation necrosis, echogenicity (homogeneous or heterogeneous), presence or absence of a central hilar structure in association with size, border and margin, etc. Theses are very interesting features for describing the lesion by US. This study based on a proper size could help us in this regard. But, the authors entirely has focused on CT findings.

**▪ Response**

We appreciate your indication of this very important issue in the present study. Endobronchial ultrasound-guided transbronchial needle aspiration (EBUS-TBNA) using convex probe EBUS is the standard technique for accessing mediastinal structures. Using convex probe EBUS, the size, border, and margin of the target lesion can be easily measured. Kurimoto *et al*. reported a dedicated classification of ultrasound findings on radial probe EBUS, with examinations performed in a perpendicular plane of the lesion by inserting a miniaturized 1.4-mm probe into the lesion. Briefly, lung lesions may be classified into three classes and six subclasses as follows: type I, homogeneous pattern (type Ia, with patent vessels and patent bronchioles; type Ib, without vessels and bronchioles); type II, hyperechoic dots and linear arcs pattern (type IIa, without vessels; type IIb, with patent vessels); and type III, heterogeneous pattern (type IIIa, with hyperechoic dots and short lines; type IIIb, without hyperechoic dots and short lines). In this study, the radial probe EBUS images of 31 patients with tuberculosis identified the lesion as type 1b in 48.4% of cases and type IIa in 22.6%. Kurimoto *et al*. found that benign lesions were highly likely to be identified as type 1, and that images of tuberculosis usually presented as type Ib. Our results are in accord with the previous study of Kurimoto *et al.,* and we have added the ultrasound findings of the present study to the Methods, Results, and appendix as follows:

Method part (see page 7, lines 5 to 6)

Ultrasound image analysis was performed according to the classifications of Kurimoto *et al*. (Appendix 2).

Results part (see page 9, lines 11 to 12)

Ultrasound image analyses are summarized in Appendix 2.

Appendix 2. Ultrasound image analyses of patients with pulmonary tuberculosis.

| Type of ultrasound image analyses | | No. (%) |
| --- | --- | --- |
| I, homogeneous pattern | Ia, with patent vessels and patent bronchioles | 2 (6.5) |
|  | Ib, without vessels and bronchioles | 15 (48.4) |
| II, hyperechoic dots and linear arcs pattern | IIa, without vessels | 7 (22.6) |
|  | IIb, with patent vessels | 1 (3.2) |
| III, heterogeneous pattern | IIIa, with hyperechoic dots and short lines | 2 (6.5) |
|  | IIIb, without hyperechoic dots and short lines | 4 (12.9) |

**References**

1. Chest. 2002 Dec;122(6):1887-94.

**# Response to the comments of Reviewer 4**

**▪ Comment 1**

It's a very interesting study. It has an important number of the people included in the study and well analyzed. I hope it will be read by the health workers working as those of the study and the responsible of them.

**▪ Response**

Thank you for your kind response. We hope that our results will be helpful for healthcare workers, to make them aware of the possibility of an unexpected diagnosis of tuberculosis when performing radial probe EBUS, and to prevent unexpected exposure to *Mycobacterium tuberculosis* during the procedure.
