# Supplementary material for: Unexpected exposure to Mycobacterium tuberculosis during bronchoscopy using radial probe endobronchial ultrasound
Source: PLoS One. 2021 Jan 28;16(1):e0246371. doi: 10.1371/journal.pone.0246371 (PMC7843011; doi:10.1371/journal.pone.0246371)
Supplement: Attachment — Submitted filename: Response to reviewer comment_minor revison.docx [file pone.0246371.s005.docx]

Thank you for inviting us to revise and re-submit our manuscript for publication in *PLOS ONE*. We have addressed the concerns raised by the reviewers in a point-by-point format. The English language was also professionally checked before this resubmission of the article. All changes have been marked in red font in the revised manuscript. We hope that the changes meet with your approval.

**# Response to the comments of Reviewer 2**

**▪ Comment 1**

Incidence (e.g. replaced by proportion) because cross-sectional studies are not deigned to measure incidence.

**▪ Response**

We agree that incidence cannot be found in cross-sectional design. We have changed the description of incidence to proportion as follows:

1. See page 2, lines 5 to lines 7 and lines 19 to 20

“In this study, we examined the proportion of and factors associated with unexpected exposure to *Mycobacterium tuberculosis* during bronchoscopy using radial probe EBUS.”

“The proportion of unexpected exposure to *Mycobacterium tuberculosis* during bronchoscopy using radial probe EBUS was 3.2%.”

2. See page 4, lines 22 to page 5, lines 3

“Therefore, we performed a multicenter cross-sectional study to identify the proportion of unexpected exposure to *Mycobacterium tuberculosis* during bronchoscopy using radial probe EBUS, and to identify factors associated with an increased probability of it.”

3. See page 11, lines 11 to 13

“In this study, the proportion of unexpected exposure to *Mycobacterium tuberculosis* during radial probe EBUS bronchoscopy for PLL was 3.2%.”

4. See page 13, lines 23 to lines 24

“We found that the proportion of unexpected unexpected exposure to *Mycobacterium tuberculosis* during radial probe EBUS bronchoscopy was 3.2%.”

**▪ Comment 2**

Unexpected diagnosis of tuberculosis, because tuberculosis can mimic any disease and because it is prevalence in the country of study setting.

**▪ Response**

We absolutely agree that pulmonary tuberculosis can mimic any lung disease and that many healthcare personnel can be accidentally exposed to *Mycobacterium tuberculosis*. The reason why we had started the present study was that we had several experiences of unexpected exposure of *M. tuberculosis* during radial probe endobronchial sound in bronchoscopy suite. To make the purpose of the current study clear, we have change the term of ‘unexpected diagnosis of pulmonary tuberculosis’ to ‘unexpected exposure to *Mycobacterium tuberculosis*’ as follows:

1. See page 1, lines 1 to 2 and lines 15

“**Title:** Unexpected exposure to *Mycobacterium tuberculosis* during bronchoscopy using radial probe endobronchial ultrasound”

“**Short title:** Unexpected exposure to *Mycobacterium tuberculosis*”

2. See page 2, lines 5 to 7 and lines 19 to 20

“In this study, we examined the proportion of and factors associated with unexpected exposure to *Mycobacterium tuberculosis* during bronchoscopy using radial probe EBUS.”

“The proportion of unexpected exposure to *Mycobacterium tuberculosis* during bronchoscopy using radial probe EBUS was 3.2%.”

3. See page 4, lines 22 to page5, lines 3

“However, little is known about the unexpected exposure to *Mycobacterium tuberculosis* during radial probe EBUS bronchoscopy for PLLs when lung malignancy is initially suspected. Therefore, we performed a multicenter cross-sectional study to identify the proportion of unexpected exposure to *Mycobacterium tuberculosis* during bronchoscopy using radial probe EBUS, and to identify factors associated with an increased probability of it.”

4. See page 8, lines 14 to 16

“Multivariate logistic regression analysis was performed using factors with a *P*-value < 0.1 in the univariate analysis, to identify independent factors related to unexpected exposure to *Mycobacterium tuberculosis.*”

5. See page 10, lines 15 to 16

“Multivariate logistic regression analysis was conducted to identify independent factors associated with unexpected exposure to *Mycobacterium tuberculosis* (Table 5).”

6. See page 11, lines 11 to 13

“In this study, the proportion of unexpected exposure to *Mycobacterium tuberculosis* during radial probe EBUS bronchoscopy for PLL was 3.2%.”

7. See page 11, lines 16 to 19

“In addition, we identified several risk factors associated with an unexpected exposure to *Mycobacterium tuberculosis*, such as age and distinct CT findings (low difference in HUs, concentric cavitation, and the presence of a satellite centrilobular nodule).”

8. See page 13, lines 23 to 24

“We found that the proportion of unexpected exposure to *Mycobacterium tuberculosis* during radial probe EBUS bronchoscopy was 3.2%.”
