# Supplementary material for: Unexpected exposure to Mycobacterium tuberculosis during bronchoscopy using radial probe endobronchial ultrasound
Source: PLoS One. 2021 Jan 28;16(1):e0246371. doi: 10.1371/journal.pone.0246371 (PMC7843011; doi:10.1371/journal.pone.0246371)
Supplement: Peer review history

# Peer review: Decision Letter 0

**DOI:** 10.1371/journal.pone.0246371.r001

**Authors:** Jeong-Seon Ryu

7 Dec 2020

PONE-D-20-30959

Unexpected diagnosis of pulmonary tuberculosis during bronchoscopy using radial probe endobronchial ultrasound

PLOS ONE

Dear Dr. Eom,

Thank you for submitting your manuscript to PLOS ONE. After careful consideration, we feel that it has merit but does not fully meet PLOS ONE’s publication criteria as it currently stands. Therefore, we invite you to submit a revised version of the manuscript that addresses the points raised during the review process.

Current clinical guidelines recommend clinicians to do protection against M tuberculosis during bronchoscopy for patients suspected of having M tuberculosis or not. Author focused on unexpected diagnosis of pulmonary tuberculosis during bronchoscopy using radial probe EBUS. Therefore, it would be very helpful if they describe what the difference in risk for M tuberculosis is between routine bronchoscopy and radial probe EBUS. In addition, they need to explain the differences between the radiological finding that they suggested as a suspected M tuberculous infection and the current knowledge or the novelity of the radiological finding that they suggested.

Please ensure that your decision is justified on PLOS ONE’s publication criteria and not, for example, on novelty or perceived impact.

Please submit your revised manuscript by Jan 15 2021 11:59PM. If you will need more time than this to complete your revisions, please reply to this message or contact the journal office at plosone@plos.org. When you're ready to submit your revision, log on to https://www.editorialmanager.com/pone/ and select the 'Submissions Needing Revision' folder to locate your manuscript file.

Please include the following items when submitting your revised manuscript:

- A rebuttal letter that responds to each point raised by the academic editor and reviewer(s). You should upload this letter as a separate file labeled 'Response to Reviewers'.
- A marked-up copy of your manuscript that highlights changes made to the original version. You should upload this as a separate file labeled 'Revised Manuscript with Track Changes'.
- An unmarked version of your revised paper without tracked changes. You should upload this as a separate file labeled 'Manuscript'.

If you would like to make changes to your financial disclosure, please include your updated statement in your cover letter. Guidelines for resubmitting your figure files are available below the reviewer comments at the end of this letter.

If applicable, we recommend that you deposit your laboratory protocols in protocols.io to enhance the reproducibility of your results. Protocols.io assigns your protocol its own identifier (DOI) so that it can be cited independently in the future. For instructions see: http://journals.plos.org/plosone/s/submission-guidelines#loc-laboratory-protocols

We look forward to receiving your revised manuscript.

Kind regards,

Jeong-Seon Ryu

Academic Editor

PLOS ONE

Journal Requirements:

When submitting your revision, we need you to address these additional requirements.

1.) Please ensure that your manuscript meets PLOS ONE's style requirements, including those for file naming. The PLOS ONE style templates can be found at

https://journals.plos.org/plosone/s/file?id=wjVg/PLOSOne_formatting_sample_main_body.pdf and

https://journals.plos.org/plosone/s/file?id=ba62/PLOSOne_formatting_sample_title_authors_affiliations.pdf

2.) In the ethics statement in the manuscript and in the online submission form, please provide additional information about the patient records used in your retrospective study, including: a) whether all data were fully anonymized before you accessed them and/or whether the IRB or ethics committee waived the requirement for informed consent; b) the date range (month and year) during which patients' medical records were accessed.

3.) Thank you for stating the following in the Acknowledgments Section of your manuscript:

'This work was supported by Department of Biostatistics, Biomedical Research Institute,

Pusan National University Hospital.'

We note that you have provided funding information that is not currently declared in your Funding Statement. However, funding information should not appear in the Acknowledgments section or other areas of your manuscript. We will only publish funding information present in the Funding Statement section of the online submission form.

Please remove any funding-related text from the manuscript and let us know how you would like to update your Funding Statement. Currently, your Funding Statement reads as follows:

 'The authors received no specific funding for this work.'

Please include your amended statements within your cover letter; we will change the online submission form on your behalf.

4.) Your ethics statement should only appear in the Methods section of your manuscript. If your ethics statement is written in any section besides the Methods, please delete it from any other section.

[Note: HTML markup is below. Please do not edit.]

Reviewers' comments:

Reviewer's Responses to Questions

**Comments to the Author**

1. Is the manuscript technically sound, and do the data support the conclusions?

The manuscript must describe a technically sound piece of scientific research with data that supports the conclusions. Experiments must have been conducted rigorously, with appropriate controls, replication, and sample sizes. The conclusions must be drawn appropriately based on the data presented. 

Reviewer #1: Yes

Reviewer #2: Partly

Reviewer #3: Yes

Reviewer #4: Yes

**********

2. Has the statistical analysis been performed appropriately and rigorously? 

Reviewer #1: Yes

Reviewer #2: Yes

Reviewer #3: Yes

Reviewer #4: Yes

**********

3. Have the authors made all data underlying the findings in their manuscript fully available?

The PLOS Data policy requires authors to make all data underlying the findings described in their manuscript fully available without restriction, with rare exception (please refer to the Data Availability Statement in the manuscript PDF file). The data should be provided as part of the manuscript or its supporting information, or deposited to a public repository. For example, in addition to summary statistics, the data points behind means, medians and variance measures should be available. If there are restrictions on publicly sharing data—e.g. participant privacy or use of data from a third party—those must be specified.

Reviewer #1: Yes

Reviewer #2: Yes

Reviewer #3: Yes

Reviewer #4: Yes

**********

4. Is the manuscript presented in an intelligible fashion and written in standard English?

PLOS ONE does not copyedit accepted manuscripts, so the language in submitted articles must be clear, correct, and unambiguous. Any typographical or grammatical errors should be corrected at revision, so please note any specific errors here.

Reviewer #1: Yes

Reviewer #2: Yes

Reviewer #3: Yes

Reviewer #4: Yes

**********

5. Review Comments to the Author

Please use the space provided to explain your answers to the questions above. You may also include additional comments for the author, including concerns about dual publication, research ethics, or publication ethics. (Please upload your review as an attachment if it exceeds 20,000 characters)

Reviewer #1: I do encourage recommendations made in this article considering the risks to HCW and their families and nosocomial infection and in addition the costs of treating Tuberculosis.

The manuscript has not clearly stated objectives to guide the focus of the study.

Reviewer #2: There are some minor changes are required:

Since the country of the study setting is moderately endemic with Tuberculosis, then pulmonary tuberculosis is a major differential diagnosis in such study candidates, and unexpected diagnosis of TB is a statement that cannot be accepted.

Retrospective study is a study design applied to cohort studies while the used design is record based cross-sectional study

The conclusion is too long and has a divergent conclusion which is the recommendation to use better protection inside bronchoscopy room which is not accepted for two reasons:

1- This conclusion is not related to the aim of the study

2- Infection control measure in bronchoscopy room ideally should protect from respiratory infections including tuberculosis, in addition, the context of the study did not assess how much personnel adhered to these measure.

Reviewer #3: This study has technical and practical points regarding undiagnosed pulmonary tuberculosis by EBUS-guided biopsies.

The diagnostic yield (predictive values) of EBUS depends on the prevalence of tuberculosis in a community. The culture and PCR are not absolutely excluding the diagnosis of TB and sometimes the clinician must rely on histopathologic description of tissue.

Regarding the risk of transmission, when a patient is sputum-negative case for AFB, the risk of transmission during the procedure is not really very high, although the broncoscopist should be aware of transmission-based precautions.

As a clinician I am eager to hear about the sonographic features of tuberculosis and mimicking process by EBUS including coagulation necrosis, echogenicity (homogeneous or heterogeneous), presence or absence of a central hilar structure in association with size, border and margin, etc. Theses are very interesting features for describing the lesion by US. This study based on a proper size could help us in this regard. But, the authors entirely has focused on CT findings.

Reviewer #4: It's a very interesting study. It has an important number of the people included in the study and well analyzed. I hope it will be read by the health workers working as those of the study and the responsible of them.

**********

6. PLOS authors have the option to publish the peer review history of their article (what does this mean?). If published, this will include your full peer review and any attached files.

If you choose “no”, your identity will remain anonymous but your review may still be made public.

**Do you want your identity to be public for this peer review?** For information about this choice, including consent withdrawal, please see our Privacy Policy.

Reviewer #1: **Yes: **Nonkqubela Bantubani

Reviewer #2: **Yes: **Layth Al-Salihi

Reviewer #3: **Yes: **Ilad Alavi Darazam

Reviewer #4: No

[NOTE: If reviewer comments were submitted as an attachment file, they will be attached to this email and accessible via the submission site. Please log into your account, locate the manuscript record, and check for the action link "View Attachments". If this link does not appear, there are no attachment files.]

While revising your submission, please upload your figure files to the Preflight Analysis and Conversion Engine (PACE) digital diagnostic tool, https://pacev2.apexcovantage.com/. PACE helps ensure that figures meet PLOS requirements. To use PACE, you must first register as a user. Registration is free. Then, login and navigate to the UPLOAD tab, where you will find detailed instructions on how to use the tool. If you encounter any issues or have any questions when using PACE, please email PLOS at figures@plos.org. Please note that Supporting Information files do not need this step.

# Author comment: Author response to Decision Letter 0

**DOI:** 10.1371/journal.pone.0246371.r002

31 Dec 2020

Thank you for inviting us to revise and re-submit our manuscript for publication in PLOS ONE. We have addressed the concerns raised by the reviewers in a point-by-point format. The English language was also professionally checked before this resubmission of the article. All changes have been marked in red font in the revised manuscript. We hope that the changes meet with your approval.

# Response to the comments of Editor

▪ Comment 1

Current clinical guidelines recommend clinicians to do protection against M tuberculosis during bronchoscopy for patients suspected of having M tuberculosis or not. Therefore, it would be very helpful if they describe what the difference in risk for M tuberculosis is between routine bronchoscopy and radial probe EBUS.

▪ Response

We agree that a description of the difference in risk factors between conventional bronchoscopy and radial probe endobronchial ultrasound (EBUS) is important for preventing healthcare workers from acquiring pulmonary tuberculosis in the bronchoscopy suite. In general, radial probe EBUS is performed for histological confirmation when lung cancer is clinically suspected through a chest CT scan. Accordingly, most radial probe EBUS results are confirmed as lung malignancy, but a few cases are unexpectedly diagnosed as pulmonary tuberculosis instead of lung cancer. Previous studies demonstrated rates of 3.9–11% for unexpected pulmonary tuberculosis diagnosis on radial probe EBUS [1,2]. However, the current guidelines recommend that bronchoscopists use an N95 mask or higher-grade respiratory precaution when mycobacterial infection is suspected [3].

We found that unexpected diagnosis of tuberculosis on radial probe EBUS is associated with a young patient age, the presence of satellite nodules, the presence of concentric cavitation, and a low Hounsfield unit difference between the pre- and post-enhanced CT. Most patients who received radial probe EBUS had a small lung nodule less than 3 cm, and accordingly, the unexpected diagnosis of pulmonary tuberculosis showed certain differences in risk factors between conventional bronchoscopy and radial probe EBUS. We previously published an article on “Exposure to Mycobacterium tuberculosis during conventional bronchoscopy in patients with unexpected tuberculosis”, and found that the presence of anthracofibrosis, bronchiectasis, or atelectasis on chest CT were independently associated with unexpected pulmonary tuberculosis [4]. To clarify the differences in risk factors associated with the unexpected diagnosis of pulmonary tuberculosis, we have modified the Discussion section as follows: 

1. See page 11, lines 8 to 11

“Previously, we found that pulmonary tuberculosis was unexpectedly diagnosed in 4.6% of patients when conventional bronchoscopy was performed, and that the risk factors associated with an unexpected diagnosis of tuberculosis on conventional bronchoscopy were anthracofibrosis, bronchiectasis, or atelectasis on chest CT [2].”

2. See page 11, lines 13 to 16

“Our results indicate that healthcare workers in the bronchoscopy suite could be accidentally exposed to Mycobacterium tuberculosis during radial probe EBUS if they do not take high-grade respiratory precautions, such as using an N95 particulate respirator.”

References

1. Am J Rrespir Crit Care Med. 2015; 192(4): 468-476.

2. Chest. 2006; 129(1): 147-150.

3. Chest. 2005; 128(3): 1742-1755.

4. PloS one. 2016; 11(5): e0156385.

▪ Comment 2

In addition, they need to explain the differences between the radiological finding that they suggested as a suspected M tuberculous infection and the current knowledge or the novelity of the radiological finding that they suggested.

▪ Response

Previous radiological studies found that the incidental discovery of pulmonary tuberculosis is associated with the presence of centrilobular nodules, cavitation, and low HU differences between pre- and post-enhanced CT. In a similar context, we identified and confirmed the risk factors for unexpected diagnosis of pulmonary tuberculosis in a patient population who received radial probe EBUS. We believe that our results will remind bronchoscopists about the radiological risk factors for an unexpected diagnosis of pulmonary tuberculosis when they perform radial probe EBUS. 

In addition, we found that a younger age was highly related to an unexpected diagnosis of pulmonary tuberculosis. Our results indicate that patients younger than 58.5 years are more likely to have pulmonary tuberculosis than lung malignancy. We believe that international readers will consider ‘age’ as the simplest and easiest patient characteristic for predicting the possibility of incidental exposure to Mycobacterium tuberculosis in the bronchoscopy suite. To improve the clarity of our findings, we have modified the Discussion section as follows (see page 12, lines 25 to page 13, lines 4): “In a similar context, we verified that a small difference in HUs (less than 4.8), concentric cavitation, and the presence of a satellite centrilobular nodule on CT were significantly associated with unexpected exposure to Mycobacterium tuberculosis in certain patient populations who received radial probe EBUS.”

▪ Comment 3

In the ethics statement in the manuscript and in the online submission form, please provide additional information about the patient records used in your retrospective study, including: a) whether all data were fully anonymized before you accessed them and/or whether the IRB or ethics committee waived the requirement for informed consent; b) the date range (month and year) during which patients' medical records were accessed.

▪ Response

To make ethics statement clear, we have modified the Methods as follows (see page 5, lines 19 to 21): “All data were fully anonymized and the ethics committee waived the requirement for informed consent. All patients’ medical records were followed-up from the time they received radial probe EBUS to December 2019.”

▪ Comment 4

Thank you for stating the following in the Acknowledgments Section of your manuscript:

'This work was supported by Department of Biostatistics, Biomedical Research Institute,

Pusan National University Hospital.'

We note that you have provided funding information that is not currently declared in your Funding Statement. However, funding information should not appear in the Acknowledgments section or other areas of your manuscript. We will only publish funding information present in the Funding Statement section of the online submission form.

Please remove any funding-related text from the manuscript and let us know how you would like to update your Funding Statement. Currently, your Funding Statement reads as follows:

 'The authors received no specific funding for this work.'

▪ Response

We agree that the acknowledgements were a little ambiguous. The ‘Department of Biostatistics, Biomedical Research Institute’ did not provide funds, but did help with statistical analysis, especially calculation of the Youden index and receiver operating characteristics curves. We have changed the Acknowledgements section as follows (see page 17, lines 3 to 4): “The Department of Biostatistics, Biomedical Research Institute, Pusan National University Hospital provided help with the statistical analysis performed in the present study” 

# Response to the comments of Reviewer 1

▪ Comment 1

The manuscript has not clearly stated objectives to guide the focus of the study.

▪ Response

We agree that the description of the objectives of the present study was somewhat unclear. In general, radial probe endobronchial ultrasound (EBUS) is performed for histological diagnosis when lung cancer is clinically suspected on CT scan. Accordingly, most radial probe EBUS results are confirmed as lung malignancy, although a few cases are unexpectedly diagnosed as pulmonary tuberculosis instead of lung cancer. Previous studies demonstrated rates of 4–11% for unexpected pulmonary tuberculosis diagnosis on radial probe EBUS, irrespective of the prevalence of tuberculosis [1-4]. However, the current guideline recommends that bronchoscopists use an N95 mask or higher-grade respiratory precaution only when mycobacterial infection is suspected clinically or radiologically [5]. 

We previously published an article on “Exposure to Mycobacterium tuberculosis during conventional bronchoscopy in patients with unexpected tuberculosis”, which revealed that the presence of anthracofibrosis, bronchiectasis, or atelectasis on CT scan were independently associated with unexpected diagnosis of pulmonary tuberculosis [6]. However, there is little known about the unexpected diagnosis of pulmonary tuberculosis during peripheral bronchoscopy involving techniques such as radial probe EBUS, virtual bronchoscopy navigation, or electromagnetic navigation bronchoscopy in patients with a small lung nodule. After the introduction of the National Lung Screening Trial (NLST) [7], the number of patients with small nodules on low-dose screening CT abruptly increased. Consequently, the use of peripheral bronchoscopy for the pathologic diagnosis of small nodules also increased. We therefore believe that it is important for pulmonary physicians to know how many patients are unexpectedly diagnosed with pulmonary tuberculosis on radial probe EBUS, and to know the relevant factors associated with its occurrence and how to prevent infection. Our results should inform and remind healthcare workers of the possibility of unexpected exposure to Mycobacterium tuberculosis when performing bronchoscopy using radial probe EBUS. To further clarify our objectives, we have modified the Background section as follows (see page 4, lines 22 to see page 5, lines 3): “However, little is known about the unexpected diagnosis of pulmonary tuberculosis during radial probe EBUS bronchoscopy for PLLs when lung malignancy is initially suspected. Therefore, we performed a multicenter cross-sectional study to identify the incidence of unexpected diagnoses of pulmonary tuberculosis and the factors associated with an increased probability of it during bronchoscopy using radial probe EBUS.”

References

1. Am J Rrespir Crit Care Med. 2015; 192(4): 468-476.

2. Chest. 2006; 129(1): 147-150.

3. Respirology. 2009; 14(6): 859-864.

4. Eur Respir J. 2002; 20(4): 972-974.

5. Chest. 2005; 128(3): 1742-1755.

6. PloS one. 2016; 11(5): e0156385.

7. N Engl J Med. 2011;365:395-409.

# Response to the comments of Reviewer 2

▪ Comment 1

Since the country of the study setting is moderately endemic with Tuberculosis, then pulmonary tuberculosis is a major differential diagnosis in such study candidates, and unexpected diagnosis of TB is a statement that cannot be accepted.

▪ Response

We thank you for pointing out a very important issue in the present study. This study was conducted in the Republic of Korea, which is an intermediate tuberculosis prevalence region (59/100 000 persons per year, 2019) [1], and we found that the incidence of unexpected diagnosis of pulmonary tuberculosis during bronchoscopy using endobronchial ultrasound (EBUS) was 3.2%. Previous studies on bronchoscopy using radial probe EBUS showed various rates of tuberculosis diagnosis, irrespective of the prevalence of tuberculosis. In their multicenter prospective study conducted in Japan, Oki et al. reported a 3.9% rate of tuberculosis on bronchoscopy for small lung nodules less than 3 cm [2]. Herth et al. reported results for bronchoscopy using radial probe EBUS performed in 2002 and 2006 in Germany [3,4]. Interestingly, the proportions of tuberculosis found differed considerably between 2002 and 2006 (4% vs. 11%, respectively). From previous studies, it can be deduced that the proportion of tuberculosis diagnosis in radial probe EBUS procedures varies depending of the physician’s case selection, regardless of where and when radial probe EBUS is performed. To clarify the description of the regional prevalence of tuberculosis and the unexpected diagnosis rate during radial probe EBUS, we have modified the Discussion section as follows (see page 11, lines 21 to see page 12, lines 8): “Previous studies on bronchoscopy using radial probe EBUS showed various rates of tuberculosis diagnosis ranging from 3.9–11.0%, irrespective of the prevalence of tuberculosis (Table 6). In their multicenter prospective study conducted in Japan, Oki et al. reported a 3.9% rate of tuberculosis on bronchoscopy for small lung nodules less than 3 cm [9], while Herth et al. reported results for bronchoscopy using radial probe EBUS performed in 2002 and 2006 in Germany [12,14], and found that the proportions of tuberculosis were quite different between the two years (4% vs. 11%, respectively). Our results suggest that the proportion of tuberculosis diagnosed on radial probe EBUS varies depending on the physician’s case selection, that it is not directly related to the regional prevalence of tuberculosis, and that the risk of healthcare personnel being exposed to Mycobacterium tuberculosis during bronchoscopy using radial probe EBUS is underestimated, with it being as high as that on conventional bronchoscopy.”

References

1. Korea Centers for Disease Control and Prevention; 2020:218-219. 

2. Am J Rrespir Crit Care Med. 2015; 192(4): 468-476.

3. Eur Respir J. 2002; 20(4): 972-974.

4. Chest. 2006; 129(1): 147-150.

▪ Comment 2

Retrospective study is a study design applied to cohort studies while the used design is record based cross-sectional study.

▪ Response

We agree that this study is of a cross-sectional design. Electronic medical records of all study subjects were reviewed from the time they received radial probe EBUS to December 2019. We have changed the description of the study design as follows (see page 4, lines 22 to page 5, lines 3): “Therefore, we performed a multicenter cross-sectional study to identify the incidence of unexpected diagnoses of pulmonary tuberculosis during bronchoscopy using radial probe EBUS, and to identify factors associated with an increased probability of it.”

▪ Comment 3

The conclusion is too long and has a divergent conclusion which is the recommendation to use better protection inside bronchoscopy room which is not accepted for two reasons:

1- This conclusion is not related to the aim of the study

2- Infection control measure in bronchoscopy room ideally should protect from respiratory infections including tuberculosis, in addition, the context of the study did not assess how much personnel adhered to these measure.

▪ Response

3-1. We acknowledge that there was too much information in the conclusion, which could confuse the reader. We have revised the Conclusion according to the objectives of this study as follows:

Abstract (see page 2, lines 19 to 23): “The incidence of unexpected diagnosis of tuberculosis during bronchoscopy using radial probe EBUS was 3.2%. A higher risk was independently associated with a younger age and CT findings of a small difference in HUs between pre- and post-enhancement images, concentric cavitation, and the presence of a satellite centrilobular nodule.”

Conclusions part (see page 11, lines 22 to page 14, lines 1): “We found that the incidence of unexpected diagnosis of pulmonary tuberculosis during radial probe EBUS bronchoscopy was 3.2%. A higher risk was independently associated with a younger patient age (less than 58.5 years) and distinct CT findings of a small difference in HUs between pre- and post-enhancement images (less than 4.8), concentric cavitation, and the presence of a satellite centrilobular nodule.”

3-2. . Generally, radial probe EBUS is performed when early-stage lung cancer is suspected on CT scan. The current guideline recommends that bronchoscopists use an N95 mask or higher-grade respiratory precaution only when mycobacterial infection is suspected according to clinical or radiological indications [1]. Therefore, radial probe EBUS is generally performed following standard precautions using an anti-droplet mask, gloves, and gown, without higher-grade respiratory precautions such as an N95 particulate respirator. Likewise, all the radial probe EBUS procedures investigated in our study were performed following standard respiratory precautions. Consequently, our results suggest that even if lung cancer is strongly suspected on CT scan, healthcare workers should be prepared for possible exposure to Mycobacterium tuberculosis if risk factors associated with an unexpected diagnosis of tuberculosis are present. To clarify the description of respiratory precautions, we have made changes to the Discussion as follows (see page 11, lines 13 to 16): “Our results indicate that healthcare workers in the bronchoscopy suite could be accidentally exposed to Mycobacterium tuberculosis during bronchoscopy using radial probe EBUS if high-grade respiratory precautions, such as the use of an N95 particulate respirator, are not undertaken.”

References

1. Chest. 2005; 128(3): 1742-1755.

# Response to the comments of Reviewer 3

▪ Comment 1

The diagnostic yield (predictive values) of EBUS depends on the prevalence of tuberculosis in a community. The culture and PCR are not absolutely excluding the diagnosis of TB and sometimes the clinician must rely on histopathologic description of tissue.

▪ Response

We agree that tuberculosis can also be diagnosed with compatible pathologic findings. In our study, four histologic samples showed chronic granulomatous inflammation, although there was no necrosis in the granuloma. None of these patients were considered to be suspicious for tuberculosis on initial CT scan, and all were followed up without any treatment. There were no changes in the size and characteristics of the lung lesions after more than 6 months of follow-up, and consequently all lesions were regarded as benign lesions other than tuberculosis. 

Generally, a thin bronchoscope (usually less than 4 mm in size) can be introduced near to peripheral lung lesions, and then a 1.4-mm radial EBUS probe is advanced through the working channel of the bronchoscope to precisely identify the peripheral lung lesion. Therefore, specimens for culture and PCR are directly collected from the lung lesion, which differs from the generally performed procedure of bronchial washing. We believe that the reliability of specimens collected for culture and PCR by radial probe EBUS was high, and that accordingly, no patient with tuberculosis was diagnosed by pathologic findings alone. To clarify the diagnostic procedure for tuberculosis, we have revised the Methods and Results as follows:

Method part (see page 8, lines 5 to 7)

“and 3) compatible histological findings, such as chronic granulomatous inflammation with clinicoradiological correlations.”

Results part (see page 9, lines 17 to 23)

“In four patients with negative results for both culture and PCR, the histologic samples showed chronic granulomatous inflammation without necrosis. Because there was no clinical or radiological evidence of tuberculosis, all these patients were followed-up without any treatment. There were no changes in the size and characteristics of the lung lesions after more than 6 months of follow-up, and consequently, all the lesions were regarded as benign lesions other than tuberculosis.”

▪ Comment 2

Regarding the risk of transmission, when a patient is sputum-negative case for AFB, the risk of transmission during the procedure is not really very high, although the bronchoscopist should be aware of transmission-based precautions.

▪ Response

We agree that the risk of tuberculosis transmission is low if a sputum acid-fast bacilli smear is negative. Unfortunately, a sputum acid-fast bacilli smear was not routinely obtained from the study patients as they were initially suspected to have a lung malignancy. Generally, patients with a small tuberculosis lesion, such as all the subjects in the present study, have low infectivity; however, the bronchoscopy procedure produces lots of respiratory droplets. We believe that healthcare workers should be aware of the risk and protect themselves from any possibility of tuberculosis infection. The relationship between the degree of infectivity and peripheral lung lesions should be verified in a future prospective study, and we have added a discussion on infectivity to the Discussion section as follows (see page 13, lines 13 to 18): “Finally, the degree of infectivity associated with small peripheral tuberculous lesions is unclear. Generally, patients with a small tuberculosis lesion have low infectivity; however, the bronchoscopy procedure produces lots of respiratory droplets. We believe that healthcare workers should be aware of the risk and protect themselves from any possibility of tuberculosis infection during bronchoscopy with radial probe EBUS.”

▪ Comment 3

As a clinician I am eager to hear about the sonographic features of tuberculosis and mimicking process by EBUS including coagulation necrosis, echogenicity (homogeneous or heterogeneous), presence or absence of a central hilar structure in association with size, border and margin, etc. Theses are very interesting features for describing the lesion by US. This study based on a proper size could help us in this regard. But, the authors entirely has focused on CT findings.

▪ Response

We appreciate your indication of this very important issue in the present study. Endobronchial ultrasound-guided transbronchial needle aspiration (EBUS-TBNA) using convex probe EBUS is the standard technique for accessing mediastinal structures. Using convex probe EBUS, the size, border, and margin of the target lesion can be easily measured. Kurimoto et al. reported a dedicated classification of ultrasound findings on radial probe EBUS, with examinations performed in a perpendicular plane of the lesion by inserting a miniaturized 1.4-mm probe into the lesion. Briefly, lung lesions may be classified into three classes and six subclasses as follows: type I, homogeneous pattern (type Ia, with patent vessels and patent bronchioles; type Ib, without vessels and bronchioles); type II, hyperechoic dots and linear arcs pattern (type IIa, without vessels; type IIb, with patent vessels); and type III, heterogeneous pattern (type IIIa, with hyperechoic dots and short lines; type IIIb, without hyperechoic dots and short lines). In this study, the radial probe EBUS images of 31 patients with tuberculosis identified the lesion as type 1b in 48.4% of cases and type IIa in 22.6%. Kurimoto et al. found that benign lesions were highly likely to be identified as type 1, and that images of tuberculosis usually presented as type Ib. Our results are in accord with the previous study of Kurimoto et al., and we have added the ultrasound findings of the present study to the Methods, Results, and appendix as follows: 

Method part (see page 7, lines 5 to 6)

Ultrasound image analysis was performed according to the classifications of Kurimoto et al. (Appendix 2).

Results part (see page 9, lines 11 to 12)

Ultrasound image analyses are summarized in Appendix 2.

Appendix 2. Ultrasound image analyses of patients with pulmonary tuberculosis.

Type of ultrasound image analyses No. (%)

I, homogeneous pattern Ia, with patent vessels and patent bronchioles 2 (6.5)

 Ib, without vessels and bronchioles 15 (48.4)

II, hyperechoic dots and linear arcs pattern IIa, without vessels 7 (22.6)

 IIb, with patent vessels 1 (3.2)

III, heterogeneous pattern IIIa, with hyperechoic dots and short lines 2 (6.5)

 IIIb, without hyperechoic dots and short lines 4 (12.9)

References

1. Chest. 2002 Dec;122(6):1887-94. 

# Response to the comments of Reviewer 4

▪ Comment 1

It's a very interesting study. It has an important number of the people included in the study and well analyzed. I hope it will be read by the health workers working as those of the study and the responsible of them.

▪ Response

Thank you for your kind response. We hope that our results will be helpful for healthcare workers, to make them aware of the possibility of an unexpected diagnosis of tuberculosis when performing radial probe EBUS, and to prevent unexpected exposure to Mycobacterium tuberculosis during the procedure.

Attachment Submitted filename: Response to reviewer comment.docx Click here for additional data file.

# Peer review: Decision Letter 1

**DOI:** 10.1371/journal.pone.0246371.r003

**Authors:** Jeong-Seon Ryu

15 Jan 2021

PONE-D-20-30959R1

Unexpected diagnosis of pulmonary tuberculosis during bronchoscopy using radial probe endobronchial ultrasound

PLOS ONE

Dear Dr. Eom,

Thank you for submitting your manuscript to PLOS ONE. After careful consideration, we feel that it has merit but does not fully meet PLOS ONE’s publication criteria as it currently stands. Therefore, we invite you to submit a revised version of the manuscript that addresses the points raised during the review process.

==============================

They have addressed some comments raised from reviewers. However, they need to repond to questions from a reviewer.

==============================

Please submit your revised manuscript by Mar 01 2021 11:59PM. If you will need more time than this to complete your revisions, please reply to this message or contact the journal office at plosone@plos.org. When you're ready to submit your revision, log on to https://www.editorialmanager.com/pone/ and select the 'Submissions Needing Revision' folder to locate your manuscript file.

Please include the following items when submitting your revised manuscript:

- A rebuttal letter that responds to each point raised by the academic editor and reviewer(s). You should upload this letter as a separate file labeled 'Response to Reviewers'.
- A marked-up copy of your manuscript that highlights changes made to the original version. You should upload this as a separate file labeled 'Revised Manuscript with Track Changes'.
- An unmarked version of your revised paper without tracked changes. You should upload this as a separate file labeled 'Manuscript'.

If you would like to make changes to your financial disclosure, please include your updated statement in your cover letter. Guidelines for resubmitting your figure files are available below the reviewer comments at the end of this letter.

If applicable, we recommend that you deposit your laboratory protocols in protocols.io to enhance the reproducibility of your results. Protocols.io assigns your protocol its own identifier (DOI) so that it can be cited independently in the future. For instructions see: http://journals.plos.org/plosone/s/submission-guidelines#loc-laboratory-protocols

We look forward to receiving your revised manuscript.

Kind regards,

Jeong-Seon Ryu

Academic Editor

PLOS ONE

Additional Editor Comments (if provided):

They have to respond to some comments from a reviewer.

[Note: HTML markup is below. Please do not edit.]

Reviewers' comments:

Reviewer's Responses to Questions

**Comments to the Author**

1. If the authors have adequately addressed your comments raised in a previous round of review and you feel that this manuscript is now acceptable for publication, you may indicate that here to bypass the “Comments to the Author” section, enter your conflict of interest statement in the “Confidential to Editor” section, and submit your "Accept" recommendation.

Reviewer #2: All comments have been addressed

Reviewer #3: All comments have been addressed

**********

2. Is the manuscript technically sound, and do the data support the conclusions?

The manuscript must describe a technically sound piece of scientific research with data that supports the conclusions. Experiments must have been conducted rigorously, with appropriate controls, replication, and sample sizes. The conclusions must be drawn appropriately based on the data presented. 

Reviewer #2: Yes

Reviewer #3: Yes

**********

3. Has the statistical analysis been performed appropriately and rigorously? 

Reviewer #2: Yes

Reviewer #3: N/A

**********

4. Have the authors made all data underlying the findings in their manuscript fully available?

The PLOS Data policy requires authors to make all data underlying the findings described in their manuscript fully available without restriction, with rare exception (please refer to the Data Availability Statement in the manuscript PDF file). The data should be provided as part of the manuscript or its supporting information, or deposited to a public repository. For example, in addition to summary statistics, the data points behind means, medians and variance measures should be available. If there are restrictions on publicly sharing data—e.g. participant privacy or use of data from a third party—those must be specified.

Reviewer #2: Yes

Reviewer #3: Yes

**********

5. Is the manuscript presented in an intelligible fashion and written in standard English?

PLOS ONE does not copyedit accepted manuscripts, so the language in submitted articles must be clear, correct, and unambiguous. Any typographical or grammatical errors should be corrected at revision, so please note any specific errors here.

Reviewer #2: Yes

Reviewer #3: Yes

**********

6. Review Comments to the Author

Please use the space provided to explain your answers to the questions above. You may also include additional comments for the author, including concerns about dual publication, research ethics, or publication ethics. (Please upload your review as an attachment if it exceeds 20,000 characters)

Reviewer #2: Thanks for authors for revising the manuscript, they addressed many issues required by the reviewers, still they need to revise two terms:

1- Incidence (e.g. replaced by proportion) because cross-sectional studies are not deigned to measure incidence

2- Unexpected diagnosis of tuberculosis, because tuberculosis can mimic any disease and because it is prevalence in the country of study setting

Reviewer #3: (No Response)

**********

7. PLOS authors have the option to publish the peer review history of their article (what does this mean?). If published, this will include your full peer review and any attached files.

If you choose “no”, your identity will remain anonymous but your review may still be made public.

**Do you want your identity to be public for this peer review?** For information about this choice, including consent withdrawal, please see our Privacy Policy.

Reviewer #2: **Yes: **Layth Al-Salihi

Reviewer #3: **Yes: **Dr. Ilad Alavi Darazam

[NOTE: If reviewer comments were submitted as an attachment file, they will be attached to this email and accessible via the submission site. Please log into your account, locate the manuscript record, and check for the action link "View Attachments". If this link does not appear, there are no attachment files.]

While revising your submission, please upload your figure files to the Preflight Analysis and Conversion Engine (PACE) digital diagnostic tool, https://pacev2.apexcovantage.com/. PACE helps ensure that figures meet PLOS requirements. To use PACE, you must first register as a user. Registration is free. Then, login and navigate to the UPLOAD tab, where you will find detailed instructions on how to use the tool. If you encounter any issues or have any questions when using PACE, please email PLOS at figures@plos.org. Please note that Supporting Information files do not need this step.

# Author comment: Author response to Decision Letter 1

**DOI:** 10.1371/journal.pone.0246371.r004

16 Jan 2021

Thank you for inviting us to revise and re-submit our manuscript for publication in PLOS ONE. We have addressed the concerns raised by the reviewers in a point-by-point format. The English language was also professionally checked before this resubmission of the article. All changes have been marked in red font in the revised manuscript. We hope that the changes meet with your approval.

# Response to the comments of Reviewer 2

▪ Comment 1

Incidence (e.g. replaced by proportion) because cross-sectional studies are not deigned to measure incidence.

▪ Response

We agree that incidence cannot be found in cross-sectional design. We have changed the description of incidence to proportion as follows:

1. See page 2, lines 5 to lines 7 and lines 19 to 20

“In this study, we examined the proportion of and factors associated with unexpected exposure to Mycobacterium tuberculosis during bronchoscopy using radial probe EBUS.”

“The proportion of unexpected exposure to Mycobacterium tuberculosis during bronchoscopy using radial probe EBUS was 3.2%.”

2. See page 4, lines 22 to page 5, lines 3

“Therefore, we performed a multicenter cross-sectional study to identify the proportion of unexpected exposure to Mycobacterium tuberculosis during bronchoscopy using radial probe EBUS, and to identify factors associated with an increased probability of it.”

3. See page 11, lines 11 to 13

“In this study, the proportion of unexpected exposure to Mycobacterium tuberculosis during radial probe EBUS bronchoscopy for PLL was 3.2%.”

4. See page 13, lines 23 to lines 24

“We found that the proportion of unexpected unexpected exposure to Mycobacterium tuberculosis during radial probe EBUS bronchoscopy was 3.2%.”

▪ Comment 2

Unexpected diagnosis of tuberculosis, because tuberculosis can mimic any disease and because it is prevalence in the country of study setting.

▪ Response

We absolutely agree that pulmonary tuberculosis can mimic any lung disease and that many healthcare personnel can be accidentally exposed to Mycobacterium tuberculosis. The reason why we had started the present study was that we had several experiences of unexpected exposure of M. tuberculosis during radial probe endobronchial sound in bronchoscopy suite. To make the purpose of the current study clear, we have change the term of ‘unexpected diagnosis of pulmonary tuberculosis’ to ‘unexpected exposure to Mycobacterium tuberculosis’ as follows: 

1. See page 1, lines 1 to 2 and lines 15

“Title: Unexpected exposure to Mycobacterium tuberculosis during bronchoscopy using radial probe endobronchial ultrasound”

“Short title: Unexpected exposure to Mycobacterium tuberculosis”

2. See page 2, lines 5 to 7 and lines 19 to 20

“In this study, we examined the proportion of and factors associated with unexpected exposure to Mycobacterium tuberculosis during bronchoscopy using radial probe EBUS.”

“The proportion of unexpected exposure to Mycobacterium tuberculosis during bronchoscopy using radial probe EBUS was 3.2%.”

3. See page 4, lines 22 to page5, lines 3

“However, little is known about the unexpected exposure to Mycobacterium tuberculosis during radial probe EBUS bronchoscopy for PLLs when lung malignancy is initially suspected. Therefore, we performed a multicenter cross-sectional study to identify the proportion of unexpected exposure to Mycobacterium tuberculosis during bronchoscopy using radial probe EBUS, and to identify factors associated with an increased probability of it.”

4. See page 8, lines 14 to 16

“Multivariate logistic regression analysis was performed using factors with a P-value < 0.1 in the univariate analysis, to identify independent factors related to unexpected exposure to Mycobacterium tuberculosis.”

5. See page 10, lines 15 to 16

“Multivariate logistic regression analysis was conducted to identify independent factors associated with unexpected exposure to Mycobacterium tuberculosis (Table 5).”

6. See page 11, lines 11 to 13

“In this study, the proportion of unexpected exposure to Mycobacterium tuberculosis during radial probe EBUS bronchoscopy for PLL was 3.2%.”

7. See page 11, lines 16 to 19

“In addition, we identified several risk factors associated with an unexpected exposure to Mycobacterium tuberculosis, such as age and distinct CT findings (low difference in HUs, concentric cavitation, and the presence of a satellite centrilobular nodule).”

8. See page 13, lines 23 to 24

“We found that the proportion of unexpected exposure to Mycobacterium tuberculosis during radial probe EBUS bronchoscopy was 3.2%.”

Attachment Submitted filename: Response to reviewer comment_minor revison.docx Click here for additional data file.

# Editor report: Decision Letter 2

**DOI:** 10.1371/journal.pone.0246371.r005

**Authors:** Jeong-Seon Ryu

19 Jan 2021

Unexpected exposure to Mycobacterium tuberculosis during bronchoscopy using radial probe endobronchial ultrasound

PONE-D-20-30959R2

Dear Dr. Eom,

We’re pleased to inform you that your manuscript has been judged scientifically suitable for publication and will be formally accepted for publication once it meets all outstanding technical requirements.

Within one week, you’ll receive an e-mail detailing the required amendments. When these have been addressed, you’ll receive a formal acceptance letter and your manuscript will be scheduled for publication.

An invoice for payment will follow shortly after the formal acceptance. To ensure an efficient process, please log into Editorial Manager at http://www.editorialmanager.com/pone/, click the 'Update My Information' link at the top of the page, and double check that your user information is up-to-date. If you have any billing related questions, please contact our Author Billing department directly at authorbilling@plos.org.

If your institution or institutions have a press office, please notify them about your upcoming paper to help maximize its impact. If they’ll be preparing press materials, please inform our press team as soon as possible -- no later than 48 hours after receiving the formal acceptance. Your manuscript will remain under strict press embargo until 2 pm Eastern Time on the date of publication. For more information, please contact onepress@plos.org.

Kind regards,

Jeong-Seon Ryu

Academic Editor

PLOS ONE

Additional Editor Comments (optional):

It is acceptable.

Reviewers' comments:

# Editor report: Acceptance letter

**DOI:** 10.1371/journal.pone.0246371.r006

**Authors:** Jeong-Seon Ryu

21 Jan 2021

PONE-D-20-30959R2 

Unexpected exposure to *Mycobacterium tuberculosis* during bronchoscopy using radial probe endobronchial ultrasound 

Dear Dr. Eom:

I'm pleased to inform you that your manuscript has been deemed suitable for publication in PLOS ONE. Congratulations! Your manuscript is now with our production department. 

If your institution or institutions have a press office, please let them know about your upcoming paper now to help maximize its impact. If they'll be preparing press materials, please inform our press team within the next 48 hours. Your manuscript will remain under strict press embargo until 2 pm Eastern Time on the date of publication. For more information please contact onepress@plos.org.

If we can help with anything else, please email us at plosone@plos.org. 

Thank you for submitting your work to PLOS ONE and supporting open access. 

Kind regards, 

PLOS ONE Editorial Office Staff

on behalf of

Dr. Jeong-Seon Ryu 

Academic Editor

PLOS ONE
